# Tetrabromobisphenol A effects on differentiating mouse embryonic stem cells reveals unexpected impact on immune system

**DOI:** 10.3389/fgene.2022.996826

**Published:** 2022-10-25

**Authors:** Alicia Tribondeau, Laurent M. Sachs, Nicolas Buisine

**Affiliations:** UMR7221 Molecular Physiology and Adaption, CNRS, Sorbonne Universités, Museum National d’Histoire Naturelle, Paris, France

**Keywords:** TBBPA, transcriptomics, neural development, immune system, mice ESC mouse embryonic stem cells (mESCs), system biology, nervous system

## Abstract

Tetrabromobisphenol A (TBBPA) is a potent flame retardant used in numerous appliances and a major pollutant in households and ecosystems. In vertebrates, it was shown to affect neurodevelopment, the hypothalamic-pituitary-gonadal axis and thyroid signaling, but its toxicity and modes of actions are still a matter of debate. The molecular phenotype resulting from exposure to TBBPA is only poorly described, especially at the level of transcriptome reprogramming, which further limits our understanding of its molecular toxicity. In this work, we combined functional genomics and system biology to provide a system-wide description of the transcriptomic alterations induced by TBBPA acting on differentiating mESCs, and provide potential new toxicity markers. We found that TBBPA-induced transcriptome reprogramming affect a large collection of genes loosely connected within the network of biological pathways, indicating widespread interferences on biological processes. We also found two hotspots of action: at the level of neuronal differentiation markers, and surprisingly, at the level of immune system functions, which has been largely overlooked until now. This effect is particularly strong, as terminal differentiation markers of both myeloid and lymphoid lineages are strongly reduced: the membrane T cell receptor (Cd79a, Cd79b), interleukin seven receptor (Il7r), macrophages cytokine receptor (Csf1r), monocyte chemokine receptor (Ccr2). Also, the high affinity IgE receptor (Fcer1g), a key mediator of allergic reactions, is strongly induced. Thus, the molecular imbalance induce by TBBPA may be stronger than initially realized.

## Introduction

Tetrabromobisphenol A (TBBPA) is a common flame retardant utilized in the manufacturing of furniture, plastics, textiles and electronic components (Shi et al., 2018). Due to that, TBBPA is widely spreads in the environment and has been detected in soil, sediment, dust, air and water (Sunday et al., 2022). Thus, wild fauna is exposed to this contaminant and TBBPA has been found in a variety of tissues and animals located in very different habitats. For example, in the aquatic species mud carp (*Cirrhinus molitorella*) and northern snakehead (*Ophicephalus argus*), it has been at high concentration, up to 670 ng/g lipid weight in serum animals living in a natural pond contaminated with e-wastes (Webster et al., 2009; Zeng et al., 2014) and up to 245 ng/g wet weight in muscle of animals isolated from North sea (Morris et al., 2004). Concentrations found in tissues of other species may be lower, but still significant (Johnson-Restrepo et al., 2008): up to roughly 9 ng/g (lipid weight) in bottlenose dolphin, 35 ng/g (lipid wt) in bull shark, 1.4 ng/g (lipid wt) in Atlantic sharpnose shark, and 35 ng/g (lipid wt) in harbor porpoises (Law et al., 2006). In barn owl, relatively high concentration (up to 6 ng/g of dry weight) are found in feathers (Eulaers et al., 2014), and also in South China waterbirds (He et al., 2010) where accumulation can be as high as 173 ng/g. During previous work, Zeng et al., 2016 detected at an electronic waste recycling site up to 260 ng/g TBBPA in chicken eggs, and 890 in goose eggs, thereby asking questions about the developmental toxicity. Human is no exception and exposure is mediated mainly by ingestion, inhalation or dermal contact (Abdallah et al., 2015). Not surprisingly, TBBPA has been detected in human plasma, serum, hair and breast milk (Cariou et al., 2008; Shi et al., 2013; Yu et al., 2019) leading in an increase of awareness concerning TBBPA potential toxicity, especially during infant development. An in-depth review of TBBPA physio-chemical properties, environmental concentrations, concentration found in biological samples from wild animals (including humans) and biological effects can be found in Sunday et al. (Sunday et al., 2022). The most well-known effects are related to its neuro-toxicity and its impact on reproduction and the hypothalamo pituitary gonadal axis. Three weeks old mice exposed through maternal milk of mothers fed with high doses of TBBPA results in neurological and behavioral alterations (freezing reflex and horizontal movement in open field) (Saegusa et al., 2009) (Nakajima et al., 2009). The main focus in term of TBBPA-driven endocrine disruption has been on the reproductive axis. Dams of pregnant Sprague-Dawley rats exposed to TBBPA display diffuse thyroid follicular cell and cellular hypertrophy (Saegusa et al., 2009), with a slight reduction of T_3_ levels in male offsprings. Male dams exposed during lactation also display lowered levels of T_3_ and T_4_, increased pituitary and testicular weight (Van der Ven et al., 2008). Hamers et la. report that TBBPA can act as an efficient competitor (IC50 = 31 nM) for T_4_ binding to transthyretin (Hamers et al., 2006), which may be a key step of the mechanism of action. It can also compete with the direct binding of T_3_ to the thyroid hormone receptors at a concentration when present in the concentration range going from 3 to 50 µM (Kitamura et al., 2005a). The antagonistic action of TBBPA against T_3_ was further confirmed with TRE responsive reporter assays in CHO (Sun et al., 2009) and HepG2 cells (Freitas et al., 2011). There are also reports of a weak estrogen activity of TBBPA. *In vitro*, it impacts testosterone biosynthesis (Dankers et al., 2013), resulting in apoptosis in testis and reduced sperm count. At the cellular level, spermatogonia show altered DNA damage response, cytoskeleton and cell cycle. Li et al., 2022 evaluated the postnatal effect of TBBPA on dams and found little effects on testis weight and serum testosterone levels but reduced cell proliferation resulting in smaller seminiferous tubule area decrease Sertoli cells and germ cells number. Unfortunately, they did not evaluate the reproduction success. Kitamura et al., 2005b showed an anti-estrogenic activity of TBBPA and an increase of uterine weight in *vivo* tests with mice. Others studies reported alterations of gene expression related to estrogen signaling targets, biosynthesis and metabolism in uterus in rats (Sanders et al., 2016; Hall et al., 2017). Dunnick et al. (2015; 2017) reported uterine epithelial tumors and endometrial epithelial atypical hyperplasia.

TBBPA also affect the nervous system. A high throughput toxicity test of a bank of 84 compounds identified TBBPA as a selective toxicant targeting dopaminergic neurons of midbrain-like tissues grown as organoids (Renner et al., 2021). Other studies found that TBBPA was more cytotoxic to neural stem cells than to neurons, astrocytes, or fibroblasts, and found that neural stem cell apoptosis was accompanied by increased reactive oxygen species generation and mitochondrial dysfunction (Cho et al., 2020). It was further shown *in vitro* that TBBPA depolarize the plasma membrane of rat cerebellar granule cells in cerebellar slices and primary cultures. This is mediated by ionotropic glutamate receptors and voltage-gated sodium channels (Diamandakis et al., 2019).

Importantly, as reviewed in (Zhou et al., 2020), most work has been carried out with very high TBBPA concentrations, well above the range found in biological samples. Because of this, and in order to characterize the effects of TBBPA during development, Shaojun Liang et al. (2019a) exposed mouse embryonic stem cells (mESCs) to low doses (nM range) of TBBPA for up to 28 days, and monitored phenotypic changes through transcriptome profiling by RNA-Seq and RT-qPCR. They could confirm that the expression of a few key developmental markers was altered, with a number of potential phenotypic outcomes. Unfortunately, they did not provide a system-wide description of the TBBPA effects on differentiating mESCs.

In this work, we provide an in-depth description of the system-wide dynamics of TBBPA effects on differentiating mESCs. By using functional genomics coupled to system biology, we identify complex transcriptional signatures of altered expression profiles. We not only confirm alteration of neuronal markers, but we also provide additional altered gene expression signatures which may serve as additional markers of TBBPA neurotoxicity. Surprisingly, we found that the strongest effect is on differentiation markers of the two lineages of immune cells, myeloid and lymphoid, with a consistent reduction of terminal differentiation markers. This may indicate that TBBPA effect on immune system is more potent than currently thought. In term of network dynamic, differentially expressed genes display a non-random distribution with a marked bias toward low-connectivity nodes, thus providing a rigorous and comprehensive description of the TBBPA impact on the network. Our work provide a set of candidate markers of TBBPA toxicity.

## Methods

### Data source

Raw data are available from the NCBI’s dedicated Sequence Read Archive (SRA) website, under the accession number GSE125324.

### RNA-seq reads processing

Raw reads were subject to quality controls with the FASTQC toolkit. Each library contains 21 to 22 million reads of PHREAD score ≥36. For all reads, the first 13 bp at the 5′ end were clipped because of contaminant sequencing adaptors. Preprocessed reads were mapped on the version 10 of the *Mus musculus* genome (mm10 from UCSC.genome.edu) using BOWTIE 0.12.3 (Langmead et al., 2009) with the following parameters: “-5 13 -m 1 -n 1 -L 35”. BOWTIE output was piped to a simple awk script transforming the data into BED6, further piped to the UNIX commands SORT and UNIQ to keep only non-redundant reads. Although this reduces the dynamic range of the gene expression measure, this is good practice to limit the biases induced by the PCR steps of the original library preparation protocol. Overall, mapping efficiency was higher than 75%. Removal of redundancy further reduced the mapped read count by a factor of two, resulting in a uniquely mapped and non-redundant reads count ≥8.5.10^6^.

Co-variance between experimental conditions was assessed by Principal Component Analysis (PCA): raw read counts were subjected to a variance-stabilization transformation as described in (Anders and Huber, 2010).

Gene expression call is based on models available from UCSC genome browser, and the reads count table was produced with the INSERSECTBED v2.25 software from the BEDTOOLS toolkit.

### Differential analysis

In functional genomics, probabilistic requirements and time + cost constrains always conflict. To circumvent this issue, the DESEQ software makes the assumption that genes of similar expression level display similar variance, thus artificially creating ‘fake’ biological replicates, and increase statistical power. While running three biological replicates is a standard, no biological replicates is still fairly common. This is the case of the GSE125324 datasets, where the ‘treated’ samples all lack biological replicates.

Because of this, it is not possible to compare intra-group vs. inter-group variance, and typical parametric differential expression calls (treatment vs. control) can not be carried out. DESeq could be run when no biological replicates are available, but this approach still suffers from very limited power, and many important DE genes may be missed, as acknowledged by the authors (Anders and Huber, 2010). Brute force permutation tests are also not practical because of the huge computational load, and will still fail to overcome the weakness of the experimental design (no biological replicates).

Therefore, differential expression calls were based on the simple metric |log_2_(Fold Change)| ≥ 1. It should be noted that despite being reasonable given the experimental setup, this threshold may be more permissible to spurious hits, especially when considering genes with low reads counts (Love et al., 2014). Nonetheless, MA-plots clearly shows that DE genes are not limited to genes with low read counts (Supplementary Figure S1).

### Gene ontology

Gene ontology analysis is based on GORILLA software suite (Eden et al., 2009), based on two unranked gene lists. We used the transcriptional signature available from CellKb v2.1.3 (Patil and Patil, 2022). This high-quality database aggregates manually collected and curated single-cell, bulk RNA-seq and microarray data.

### Biological networks

The network is build upon pathways extracted from the KEGG pathways database (Kyoto Encyclopedia of genes and Genomes database) (Kanehisa and Goto, 2000), with the CYTOSCAPE v3.8.2 environment (Shannon et al., 2003) all KEGG pathways containing at least one DE gene were collected with the JEPETTO plugin (Winterhalter et al., 2014) the XGMML files produced by CYTOSCAPE were parsed iteratively to fill in a n x n triangular matrix M representing the final network, where each entry M_ji_ correspond to a functional interaction between the genes i and j (adjency table). Here, nodes correspond to gene products and links are the functional connections connecting them (*e.g*. A regulates B, which acts on A and C...). Importantly, given that KEGG pathways store pathways of many different types (signaling pathways, metabolic pathways, ), the resulting network is neither a metabolic pathway nor a signaling pathways. We prefer to refer to it as a network of biological pathways. Network properties were computed with in-house scripts, in a manner similar to other published tools (*e.g*. NETWORKX), or with CYTOSCAPE.

Network layouts were computed with the “edge weighted spring-embedded” algorithm and reworked by hand to improve visual quality. Hubs are defined as nodes with high connectivity (degree k ≥ 20) (Chin et al., 2014). The degree distribution follows a power low distribution, as expected for biological networks displaying scale free and small world properties (*i.e.* short distance between randomly selected nodes compared to a random network).

Permutation tests were carried out with PYTHON scripts using RANDOM, NETWORKX and SCIPY libraries. They are run with 1,000 iterations.

## Results

### Effects of TBBPA are time and dose dependent

This work is based on the data set produced by (Liang et al., 2019b), where they exposed differentiating mESCs to 10 or 100 nM TBBPA for 4–28 days, and measured transcriptional responses by RNA-seq.

**FIGURE 1:**
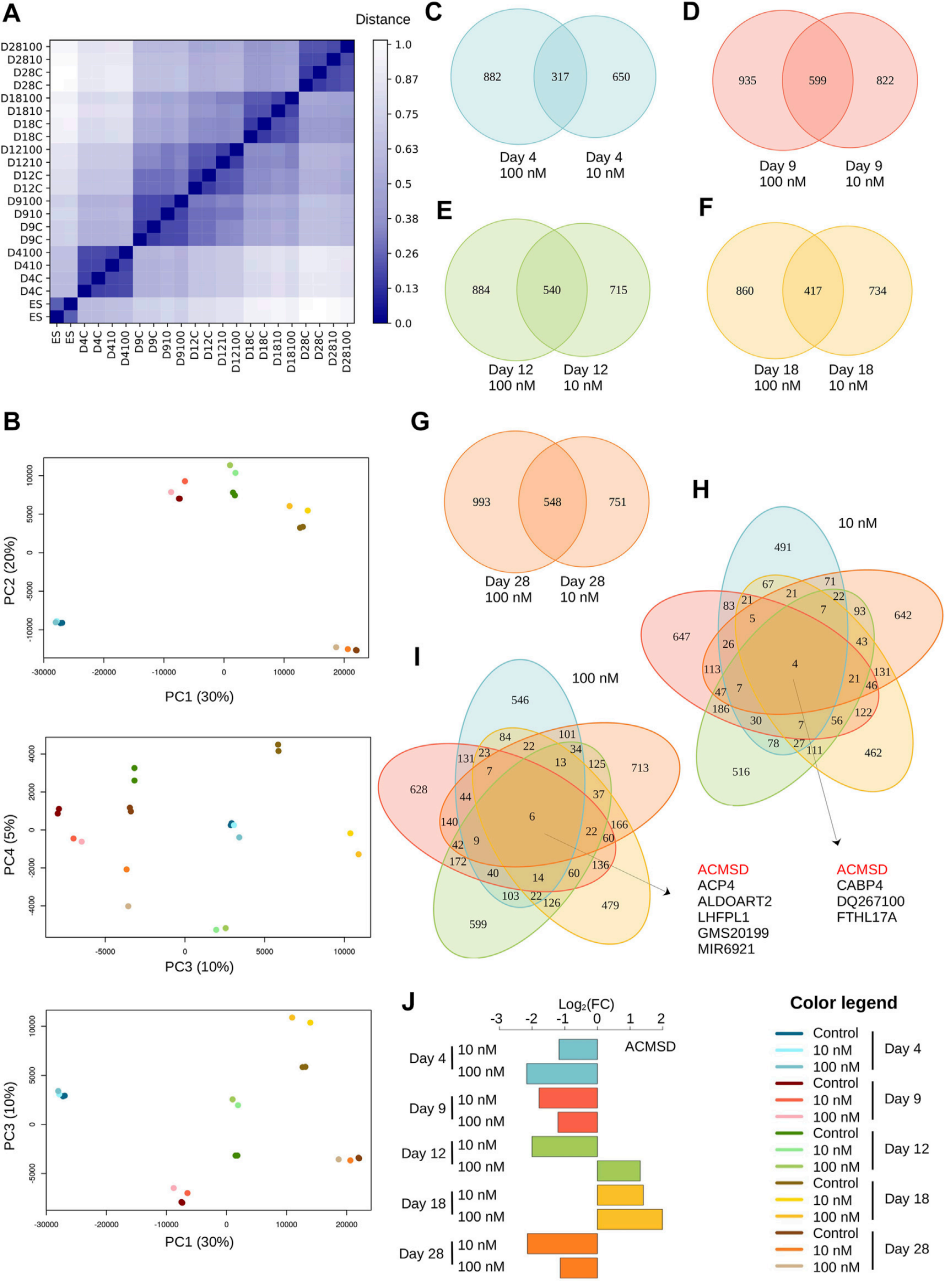
TBBPA treatment has long term effect on transcriptome. **(A)** Distance matrix between samples, shown as a heatmap. Color gradient range from dark blue for identical samples (distance = 0) to white (distance = 1). **(B)** Principal Component Analysis (PCA). Only the first four major components (45% total variance) are shown. **(C–G)** Overlap between DE genes after treatment with TBBPA 100 nM (left) or 10 nM (right), for each time point. **(H–I)** All-by-all comparison DE genes lists. **(J)** Changes of expression level (log2 scale) of ACMSD, the only gene differentially expressed in all experimental conditions.

Hierarchical clustering of normalized read count data clearly group samples by time point, suggesting that there are more differences between treatment duration compared to response to the two TBBPA concentrations (distance between experiments displayed as a heatmap, Figure 1A). Principal component analysis (PCA) further reveals that most of the total variance is explained by only four components (65% from PC1 to PC4). The main component (PC1) explains 30% of the variance and clearly corresponds to individual time points (Figure 1B). All experimental conditions are grouped by time point; that is, 10 nM and 100 nM treatment are grouped together at different time points. Only the fourth component (PC4) shows grouping according to TBBPA concentration, suggesting that this effect is quantitatively modest (5%). Therefore, most of the differences are between time point and TBBPA treatments have only a modest effect on the transcriptome output. The transcriptomic impact of TBBPA concentrations is even lower.

We identified between 967 and 1,541 genes as differentially expressed (DE) (|log_2_foldchange| ≥ 1, Supplementary Table S1) in all treatment conditions tested. This reduces to a non-redundant set of 5998 DE genes with ∼48% (2,909/5,998) found in at least one time point at both concentration, and ∼21% (1,294/5,998) in at least one time point at 10 nM and ∼29% (1795/5,998) at 100 nM. Overall, these values are well within range of similar experimental datasets reported by others, even if these studies report very different number of DE genes, varying over one range of magnitude (from 342 in Lu et al., 2021 to 3,308 in Guyot et al., 2014). We next asked whether treatment with higher dose result in stronger or faster biological responses, as could be expected from cascading effects on developmental processes. First, we find that treatment with 100 nM TBBPA tend to produce more DE genes than with 10 nM, but this is not systematic. For example, at D9 there are 1421 DE genes after 10 nM treatment (and 1,534 at 100 nM), which is higher than at D18, with 1277 DE genes at 100 nM (and 1,151 at 10 nM). Also, the number of DE genes does not increase with treatment duration (967 at D4, 1,421 at D9, but 1,255 at D12 and 1,299 at D28). Strikingly, we found only modest overlap between the set of DE genes at 10 and 100 nM treatment for each time point (*i.e.* when not considering treatment duration, Figures 1C–G). The overlap ranges from 17.1% for D4 [(882 + 317+650)*100/317 = ∼17.1%] to 25.4% for D9. This indicate that the transcriptional response induced by each TBBPA concentration is dominated by a very specific component. In other words, higher TBBPA exposure does not translate into « just » a stronger transcriptional response inclusive of responses found at lower doses. Of note, this result does not contradict the PCA analysis, which considers the expression level of all genes, while we focus here only on DE genes. We next asked how many DE genes are in common between transcriptional responses at different time points (Figures 1H,I). We found very little overlap and the vast majority of DE genes is only found at one time point, in both 10 and 100 nM treatments. Only four genes are always DE with 10 nM (*Acmsd*, *Cabp4*, *Dq267100* and *Fthl17a*) and 6 with 100 nM (*Acmsd*, *Acp4*, *Aldoart2*, *Lhfpl1*, *Gm20199* and *Mir6921*). *Acmsd* is the only gene regulated at both concentrations. The transcriptional response of all these genes, including *Acmsd*, is highly variable and can be either strongly or weakly induced or repressed, depending on treatment duration (Figure 1J). Finally, we addressed whether the small overlaps could be explained by a temporal shift of transcriptional responses driven by higher TBBPA concentrations: for example, some DE genes can potentially display the regulation pattern from a time point (e.g. D4 to D18) at 100 nM, but shifted toward longer time response at lower concentration (e.g. from D9 to D28). We found very little evidence supporting a temporal action of TBBPA, with only 5% (117/2,271) of DE genes following this trend.

Altogether, these results clearly show that exposure to different TBBPA concentrations results in contrasted transcriptional responses, with only little change of the temporal cellular responses.

### A broad and diverse set of biological pathways affected

We used standard tools and procedures to perform gene ontology analysis in order to highlight the biological processes involved. Overall, we found 552 GO terms significantly enriched in at least one condition (Supplementary Table S2). The most represented terms across experiments (*i.e.* found in at least nine out of 10 conditions) are very broad and general (level 1 or two GO terms): system process, calcium ion homeostasis, cell-cell signaling, defense response, inorganic ion transmembrane transport, ion transmembrane transport, positive regulation of cytosolic calcium ion concentration, signaling, ion transport, inflammatory response, multicellular organismal process (Supplementary Table S3) with a fair enrichment in ion transport. In general, terms are highly diverse and do not contrast any specific process or organ. For example, the term female pregnancy is enriched in five out of 10 experiments, as it is also the case for the term chemokine-mediated signaling pathway, response to lipid and hormone metabolic process. A majority of terms (325/552) are only found enriched in a single experimental condition.

The number of genes associated to each term is highly variable and range from 3 to 2,180. Among these, each individual member of a small subset 542) contribute to at least 10 different GO terms, indicating that the gene ontology representation is biased by this small gene set. In general, enriched terms are not limited to a single experimental condition but they are found in several, resulting in a limited GO contrast between experiments. This analysis clearly shows that TBBPA has a very broad and general impact on many biological functions, with only very limited contrast toward specific terms.

### Network biology to identify biological processes and makers affected by TBBPA

**FIGURE 2:**
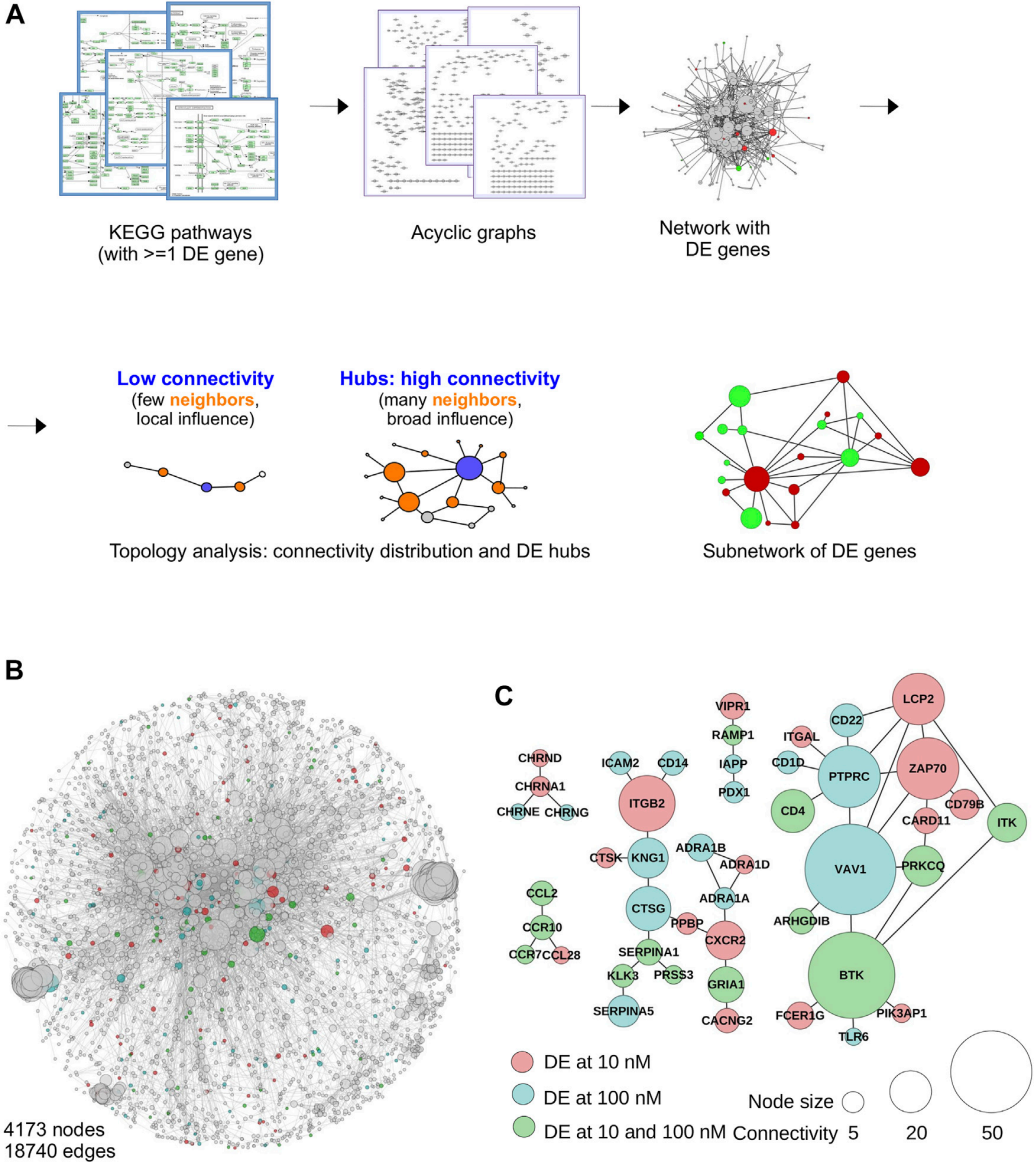
Integrative analysis of TBBPA-induced perturbations in biological networks. **(A)** Principle of network reconstruction, by combining all KEGG pathways containing at least one DE gene, and merging together individual graphs (pathways). The resulting network is then annotated with DE genes, highlighted in color. The network is then used to interrogate individual node’s connectivity and detect the influence of DE genes in the network (local vs. broad), and identify subnetworks representing focus points of action. **(B)** The reconstructed network (4,173 nodes, 18,740 edges) has the typical structure of scale free networks, with a large number of low connectivity nodes and a limited number of hubs. Connectivity is displayed as node size. DE genes are color-coded. **(C)** Subnetworks obtained after 9 days TBBPA treatment, showing all the DE genes functionally interacting with each other. Most contain only a few nodes, except much largest one.

We used the formal framework of system biology to provide a more mechanistic description of the processes involved at the cellular and molecular levels. Although this might seem counter-intuitive, data integration at a system-wide level is very efficient at highlighting unusual features or feature responses within biological networks, and thus readily pinpoint (sets of) genes of functional significance in the experimental context. To this end, we proceed in two steps: we first run a pathway analysis based on the popular KEGG pathways database (Kanehisa and Goto, 2000), and we then build a network of biological pathways for data integration. In principle (Figure 2A), the network is build by collecting and merging together all the pathways containing at least one DE gene, whether or not they are significantly enriched (Kerdivel et al., 2019; Buisine et al., 2021). This results into a network where nodes are gene products and edges (links) represent the functional interaction found in the original pathways. This approach has multiple benefits: it is gene centric and does not rely on pathway enrichment; it takes into account the fact that pathways are not independent if they share one or many gene products; it benefits from the theoretical and technical tools developed in the field of system biology. One of the most important product of this analysis is the identification of highly connected nodes, also known as hubs. The biological significance of hubs is important because affecting their expression level is expected to translate into numerous functional effects. As such, they correspond to central sensors or regulators of cellular activity (Zhu et al., 2007). Network analysis also helps identify subnetworks composed of DE genes directly connected, if they exist. These are focus points of concerted regulation within the network and certainly represent key mediators of the biological response, especially if they contain hubs. This is the rationale of our analysis: are hubs favored targets or are they avoided? Are functionally relevant subnetwork being affected?

KEGG pathways collected for network reconstruction at each experimental condition are summarized Supplementary Table S4. Only a very limited number of experimental conditions showed evidence of statistically significant pathway enrichment (based on XD-score ≥ 1). These correspond to the D9 at 10 nM and the D18 at 100 nM datasets with the pathways « renin-angiotensin system », « retinol metabolism », « tyrosine metabolism ». This apparent similarity between these two datasets is not reflected by the PCA which shows instead marked differences between time points (Figure 1B). This is in fact the result of a small number of DE genes shared between the corresponding pathways (*Lrat*, *Tpo*, *Adh1a* and *Adh1b*). This indicate that the action of TBBPA is not mediated through the coordinated regulation of entire pathways (as described in the KEGG database). Instead, this suggests that DE genes are scattered throughout numerous pathways, which fail to reach statistical significance in enrichment analysis.

The reconstructed network has a total of 4,173 nodes and 18,740 edges (Figure 2B), which is well in range of previous studies on other models (Kerdivel et al., 2019; Buisine et al., 2021). This corresponds to a total of 161 aggregated KEGG pathways containing at least one DE gene, for a subset of 733 DE genes found in the database. We found that as little as 18 DE genes involved in signaling (*Adcy7*, *Calml3*, *Fas*, *Faslg*, *Hla-doa*, *Hla-drb1*, *Hla-drb5*, *Igf1*, *Il10*, *Il6*, *Itga2*, *Mapk10*, *Nos2*, *Pik3cg*, *Pla2g10*, *Pla2g4a*, *Plcb2*, *Rac2*) contribute the most since they are found in 92 of the 161 KEGG pathways used. This is expected since biological processes often rely on shared molecular mechanisms and signaling, *e.g. Pi3k* mediates both thyroid hormone and insulin signaling (Fu et al., 2021; Byrnes et al., 2022).

Downstream network analyses will address the degree distribution of DE genes within the network and the predicted functional output of subnetworks (see sections below). Of note, even the straightforward identification of a subnetwork at individual time points already provides important functional informations. An example is shown Figure 2C, where 47 DE genes at D9 functionally interact with each other. Among them, two hubs, VAV1 and BTK, clearly stand out and readily point to immune system related functions. Close examination further reveals additional connection to immune systems: ITK, TLR6, ITGAL, CD4... Nonetheless, although powerful, this approach fails to integrate all time points together, and significant time-dependent connections may be missed.

### Most transcriptome alterations are scattered throughout the biological network

The question being asked is whether transcription alterations are randomly distributed within the network or whether preferentially affect (or avoid) hubs. It is important to note that even under-representation of hubs is of interest since they stand out more. In order to characterize the network dynamics, we carried out a topology analysis, *i.e*. we scored node connectivity and asked whether DE genes tend to be highly or poorly connected.

**FIGURE 3:**
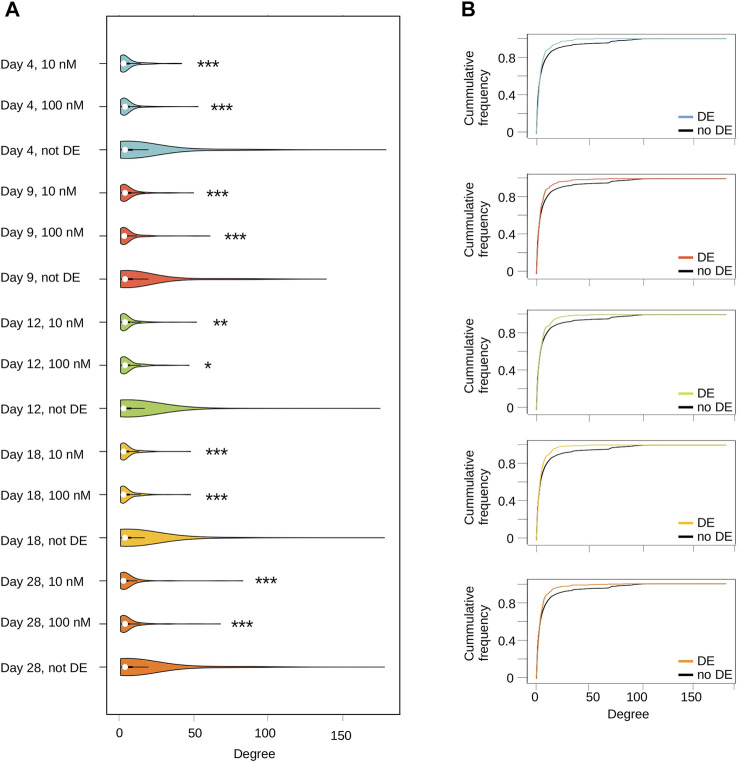
Transcriptome alterations induced by TBBPA are not randomly distributed in biological networks. **(A)** Connectivity (degree) distribution of DE genes *versus* no-DE, at each time point. * pval ≤0.05, ** pval ≤10^–2^, *** pval ≤10^–3^. **(B)** Cumulative proportion of network nodes relative to their degree (Empirical Cumulative Distribution Function). DE genes are always located above non-DE genes, indicative of a higher proportion of poorly connected nodes.

Overall, we found that DE genes connectivity is always significantly lower (on average) compared to non-DE genes (Figure 3A). This can be precisely quantified by estimating empirical cumulative distribution functions (ECDF) coupled to permutation tests (Figure 3B). In our case, this procedure provides the proportion of nodes having a connectivity less or equal than a target value ranging from one to n (n = max connectivity). For example, a population of nodes with low connectivity will have 95% of them (frequency = 0.95) with a degree less than some value (*e.g.* 10), while a population of highly connected nodes may only have a limited proportion (*e.g.* 70%, frequency = 0.7). At a connectivity value of 25, our results unambiguously show a statistically significant higher ECDF value for DE genes compare to non-DE genes in the network (Supplementary Table S5), hence indicative of a lower overall connectivity. This indicates that TBBPA act at multiple isolated targets spread within biological networks, and its effects are thus very local. As shown above, this is not associated to a clear and specific functional signature, but rather to a very diverse set of biological processes. This is an important result because this suggests that TBBPA affect diverse molecular targets, cascading into a large range of biological processes.

### Network biology uncovers alterations of nervous and immune system markers

**FIGURE 4:**
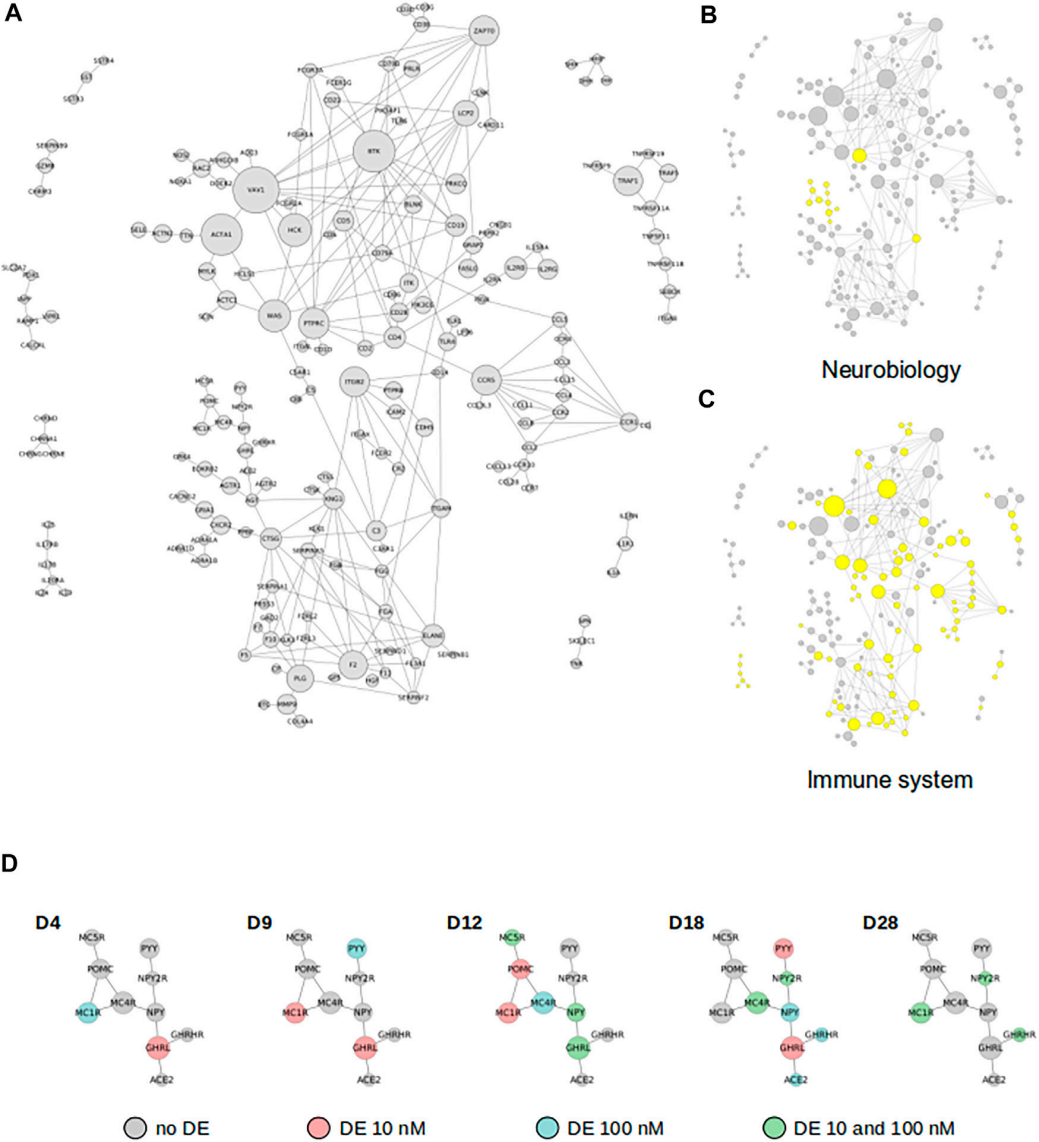
DE genes functionally cooperate with each other. **(A)** Subnetwork depicting the functional interactions between DE genes. **(B,C)** Genes related to neurobiology or immune system are highlighted in yellow, respectively. **(D)** Temporal dynamic of the subnetwork where most of genes (8 out of 10: *Pomc*, *Mc4r*, *Pyy*, *Npy2r*, *Npy*, *Ghrl*, *Ghrhr* and *Ace2*) are highly expressed in central nervous system.

A significant fraction of all DE genes is not scattered within the network but are instead directly connected to each other, forming a dense subnetwork of coordinated responses (Figure 4A). Indeed, subnetworks composed of DE genes directly connected with each other collectively participate to regulatory processes, and they are often significant mediators of biological responses. We found such subnetworks at each time point (between 5 and 12 per time point, see example Figure 2D) and they all share similar properties: between 3 and 23 DE genes and a slight enrichment of hubs. Roughly a third of all genes found in these subnetworks are shared between at least two time points, suggesting that they may functionally overlap. Two extremes are VAV1 and BTK, two hubs (k = 55 and k = 50, respectively) involved in immune system related pathways, and found in the subnetworks at all time points but one (D18, not shown). The mere presence of overlap between the subnetworks found at different time points suggest a functional and/or a temporal connection that needs to be addressed by combining DE genes lists.

By combining all DE genes, irrespective of the treatment dose or duration, we found that a significant fraction of them actually form a giant subnetwork, *i.e.* each (DE) node is directly connected to at least one other DE node (Figure 4A). Its size is slightly smaller than expected by chance (permutation test, *p* = 0.030, z-score = -1.87), which is consistent with the overall reduced connectivity of DE genes (compared to non-DE genes) within the network (see below). The subnetwork is composed of 146 nodes, including 19 hubs (degree ranging from 20 to 56, Supplementary Table S6). Remarkably, the subnetwork captures the majority of DE hubs (19/35 = 54%), a contrast well supported statistically (*p* ≤ 10^–5^, z-score = 9.27). Also, combining the datasets make the temporal coordination of gene expression more apparent, as illustrated by the successive expression of the neuro-endocrine system markers (GHRL, NPY, MC5R, MC4R, POMC, NPY2R, MC1R, ACE2, GHRHR and PYY, Supplementary Video S1). Altogether, and given the structural and functional importance of hubs, it is clear that this subnetwork captures a significant part of the molecular phenotype induced by TBBPA challenges.

Strikingly, Gene Ontology analysis (using non-subnetwork genes as background) restricted to the gene set of the subnetwork shows a very strong enrichment of biological processes related to the immune system (Table 1, top 20 GO terms). This corresponds to the main biological functions expressed collectively within the subnetwork. This signature results from a combination of GO terms like “immune response” (GO:0006955), “inflammatory response” (GO:0006954), “positive regulation of immune system process” (GO:0002684), “defense response” (GO:0006952), “immune response-activating signal transduction” (GO:0002757), “cell surface receptor signaling pathway” (GO:0007166) and “G protein-coupled receptor signaling pathway” (GO:0007186), to name a few. Even if the subnetwork contains a few genes involved in various aspects of neurobiology (*Mc5r*, *Pomc*, *Mc1r*, *Mc4r*, *Pyy*, *Npy2r*, *Npy*, *Ghrl*, *Ghrhr*, *Ace2*, *Ptprc* and *Itgam*), we found no statistical enrichment of GO terms related to neurobiology and neurodevelopment.

**TABLE 1:** Top 20 of GO terms enriched in the subnetwork. Most of them are related to immune system functions.

*p*-value	FDR q-value	Enrichment fold
2.08E-22	2.59E-18	3.43
1.18E-21	7.38E-18	4.51
6.38E-21	2.65E-17	2.18
2.17E-20	6.76E-17	1.68
3.49E-20	8.71E-17	2.96
3.56E-20	7.38E-17	3.15
4.65E-20	8.28E-17	2.3
1.71E-16	2.67E-13	1.7
5.54E-16	7.67E-13	2.38
2.8E-15	3.49E-12	3.98
3.08E-15	3.49E-12	5.48
3.89E-15	4.04E-12	3.09
4.13E-15	3.96E-12	3.69
4.27E-15	3.8E-12	2.91
6.58E-15	5.46E-12	1.93
7.24E-15	5.64E-12	2.88
9.65E-15	7.07E-12	2.69
3.07E-14	2.13E-11	3.64
4.45E-14	2.92E-11	2.93
1.4E-13	8.73E-11	2.85

**FIGURE 5:**
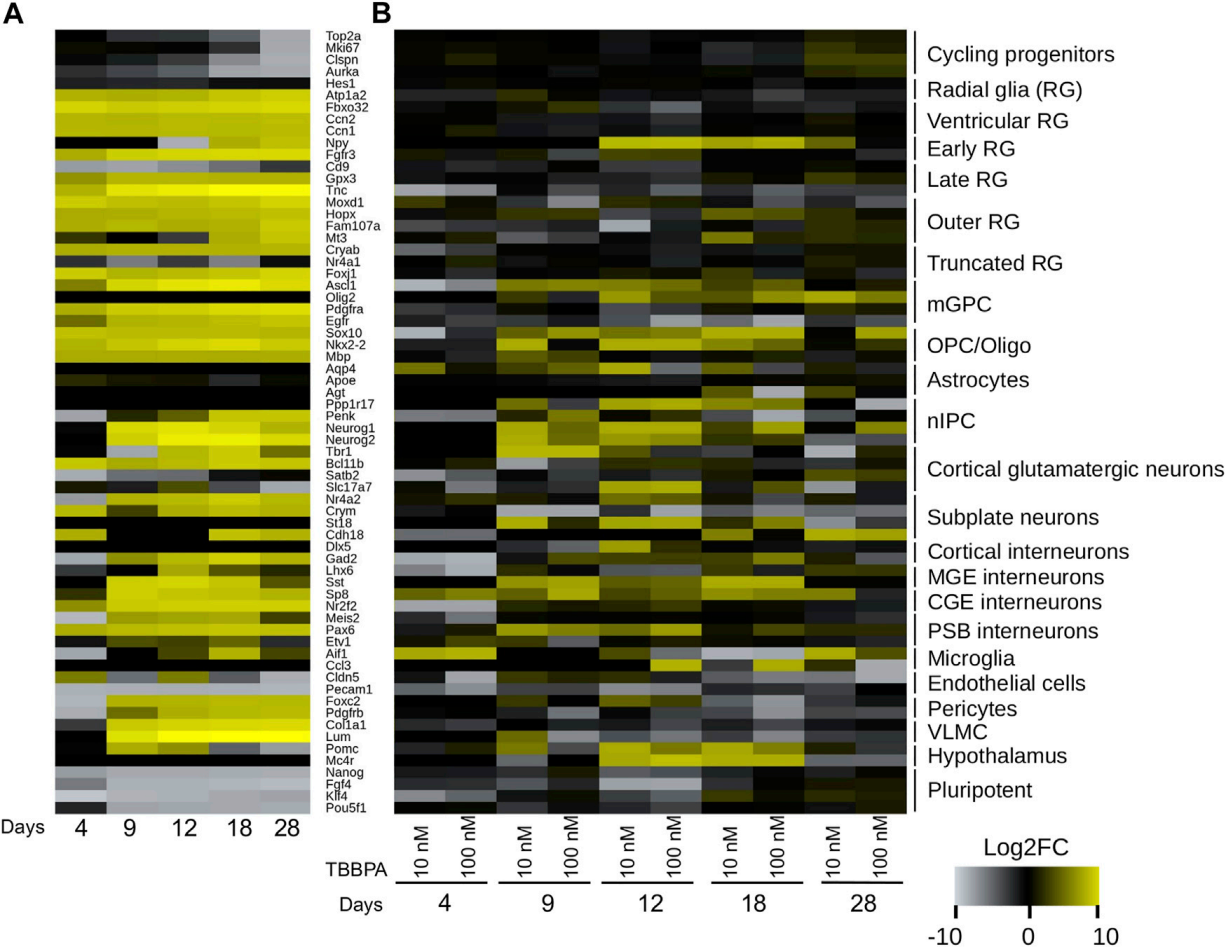
Time dependent evolution of differentiation markers. Heatmaps of expression changes (Log_2_ scale). **(A)** Spontaneous differentiation without treatment (D0 vs. D4, D9, D12, D18 and D28). **(B)** After 10 nM or 100 nM TBBPA treatment. RG: radial glia. mGPC: multipotent glial progenitor cell. nIPC: neuronal intermediate progenitor cell. MGE: medial ganglionic eminence. CGE: caudal ganglionic eminence. PSB: pallial-subpallial boundary. VLMC: leptomeningeal cell. Markers are from (Trevino et al., 2021).

We next focus on two regions of the subnetwork (Figures 4A–C), related to neuronal functions on the one hand, and the immune system on the other. The first region of interest is a group of 10 connected genes (*Mc5r*, *Pomc*, *Mc1r*, *Mc4r*, *Pyy*, *Npy2r*, *Npy*, *Ghrl*, *Ghrhr* and *Ace2*), with eight of them being strongly expressed in central nervous system (Figure 4B). They display a gradual and time dependent regulation (Figure 4D): only two to three genes are DE at D4 and D9 (*Mc1r*, *Ghrl* and *Pyy*), six genes are DE at D12 (*Mc5r*, *Pomc*, *Mc1r*, *Mc4r*, *Npy* and *Ghrl*). The transition to D18 is marked by the progressive shutdown of three genes (*Mc5r*, *Pomc* and *Mc1r*) and the regulation of four additional genes (*Pyy*, *Npy2r*, *Ghrhr* and *Ace2*). This temporal pattern, even if limited to only a small region of the subnetwork, is a strong confirmation of a TBBPA effect at the neurological level. In order to investigate this further, we addressed whether a collection of neuro-developmental markers (Trevino et al., 2021) also follow this trend. We proceed in two steps: we first describe the normal evolution of gene expression in differentiating mESCs, before documenting the impact of various TBBPA concentration at each time point. Without TBBPA treatment, mESCs show a rapid and abrupt shutdown of pluripotence markers (*Nanog*, *Fgf4*, *Klf4*, *Pou5f1*, Figure 5A), and a more gradual repression of cycling progenitors markers (*Top2a*, *Mki67*, *Clspn*, *Aurka*). Although astrocytes markers seem unaffected, this indicate that mESCs spontaneously differentiate in various neural cell populations. TBBPA has a relatively strong impact (Figure 5B) on the expression of neuronal markers (mature and immature: *St18*, *Sst*, *Sp8*, *Pax6*, *Ppp1r17*, *Neurog1/2*) and early radial glia cells (*Npy*). The differentiation markers of others cell populations also follow this dynamic but to a lesser extend: *Sox10* and *Nkx2-2* for OPC-oligodendrocytes, *Ascl1* and *Olig2* for mGPC cells. The majority of glia markers are shutdown, as opposed to neuron markers which tend to be more expressed. Oligodendrocytes and intermediate progenitor markers display more diverse regulations often going in opposite direction. These results not only confirm the known impact of TBBPA on the nervous system, they also pinpoint strong contrasts between cell-populations (neuron vs. glia).

**FIGURE 6:**
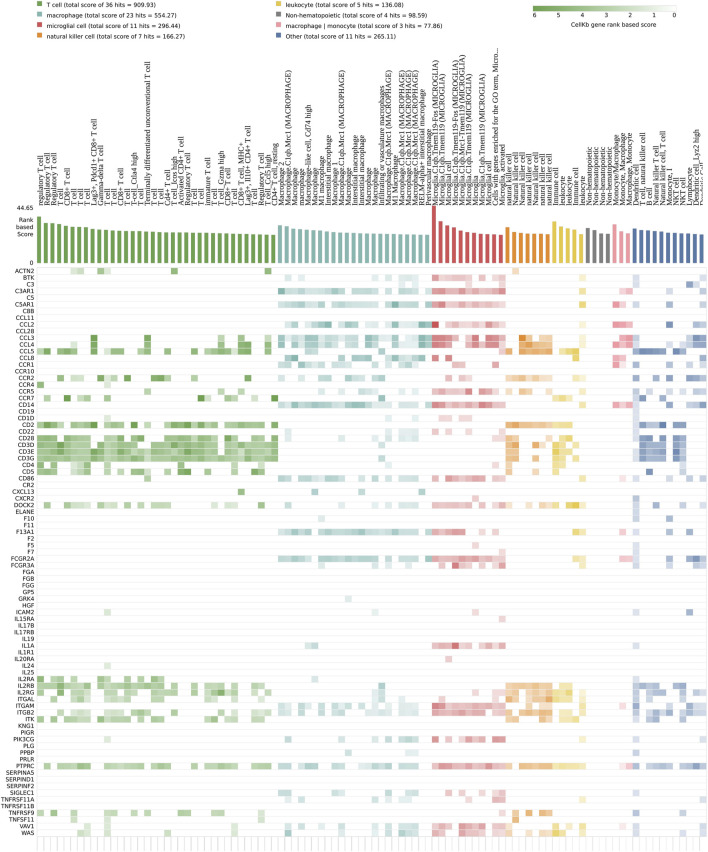
The transcriptional signature of the subnetwork corresponds to immune system functions. DE genes are compared to expression changes specific of individual immune cell types. Color code is according to the various cell lineages, where strong colors correspond to higher scores.

**FIGURE 7:**
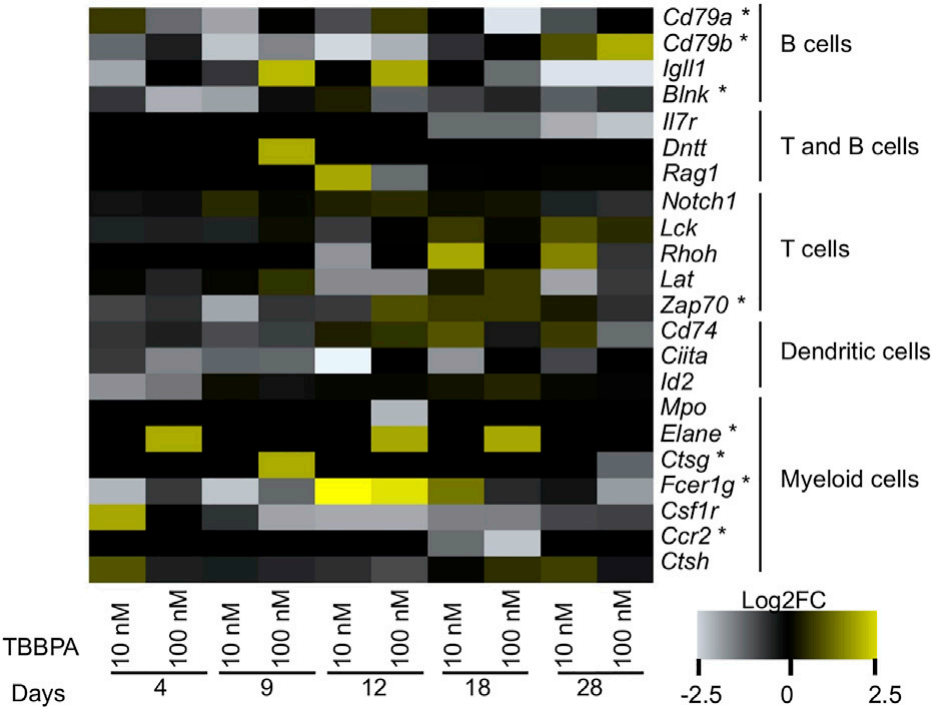
Transcriptome alterations of immune cell differentiation markers, after exposure to TBBPA. Markers are based on single cell analysis of gene expression. Genes labelled with a star (“*") belong to the subnetwork.

The second focus point correspond to the immune system (Figure 3C). The strong signature of immune system markers does not originate solely from the astrocytes and microglia cells found in the nervous system (Figure 6). In fact, a cellkb analysis of the subnetwork genes reveals a second and clearly distinct set of immune system markers found in T cells and natural killer cells lineage, and more generally in the lymphoid lineage (Figure 6, Supplementary Table S7). We find remarkable that a majority of the nodes located in the subnetwork match the transcriptional signatures of the immune cells. We next performed the same analysis as for neuro-developmental markers, and we plotted the expression value of a set genes used as markers of different cell populations (Figure 7) (Alberti-Servera et al., 2017). Strikingly, the heatmap is dominated by a lowered expression of these markers, meaning that with TBBPA, most cells fail to reach the differentiation state they would have reached without. The other remarkable feature is that all cell types are affected, both from myeloid and lymphoid cell lineages.

Altogether, this integrative analysis in differentiating mESCs clearly shows that TBBPA affects the expression pattern neural markers, and uncovers new effects on immune cells.

## Discussion

In previous work, Liang and al. Setup an *in vitro* system to address the long duration treatment effects of continuous exposure to TBBPA. To this end, they used the differentiation of mouse embryonic stem cells (mESCs) to embryoid bodies as a sensitive assay to score the toxicity of TBBPA during development. In this work, we used a post genomic analysis framework to provide a system-wide description of the functional disturbances induced by TBBPA. Although often perceived as challenging, system biology is a natural complement to functional genomics that chaperon the transition from raw results (lists of DE genes) to a biological model. Indeed, the biological interpretation of high throughput data is notoriously laborious because it produces long lists of DE genes which are difficult to make sense of spontaneously. But by providing a wide and naive view of a biological system, network analysis can readily identify “unusual” region with respect to the experimental perturbations, without functional *a priori*. Our analyses provide novel observations: we first show that response to high and low doses of TBBPA are vastly different from each other, and that a high dose does not accelerate the stereotype response of a low dose treatment. We next show that the majority of TBBPA-induced gene expression alterations are scattered throughout biological networks, although a few hubs and subnetworks stand out. TBBPA affects the expression of several markers of neural cell populations, including oligodendrocytes as well as neuronal intermediate progenitor. We also uncover a strong transcriptional impact on immune system markers, including myeloid and lymphoid lineages. The functional impact of TBBPA is therefore the expression of its cumulative toxicity at two subnetworks together with a more diffuse and widespread component, that should be addressed as a whole.

### On the challenge of making biological sense of large lists of DE genes

#### System biology magnify the functional description of transcriptomic datasets

Functional genomics (RNA-seq and microarray analyses) often produce very long lists of genes of interest, of the order of a few thousand per experiment, which far exceeds the processing capabilities of a human brain and prevent the conceptual modeling of the processes under study. Gene ontology and pathway analysis are very popular approaches used to reduce the dimensionality of the datasets and to provide a more biologically-sound summary of complex biological responses. This is made possible with knowledge databases organized according to different paradigms: the Gene Ontology database (Ashburner et al., 2000), the KEGG pathway database (Kanehisa and Goto, 2000) and others. Gene ontology is modeled through hierarchical relationships between “GO terms”, each associated to a set of genes, and addressed with a controlled vocabulary, while KEGG pathway are user defined and curated collections of molecular interactions called “pathways”.

Fundamentally, the approaches are based on a measure of enrichment of DE genes compared to lists of genes known to be involved in specific biological processes. In the case of the popular tool GOrilla, enrichment is defined as E = (b/n)/(B/N), where b is the number of DE genes of the categories, n is the total number of DE genes, B is the total number of genes associated with a specific GO term and N is the total number of genes (Eden et al., 2009). In this context, it is clear that strong biological responses (b high) channeled through a limited number of biological processes would result in a high b/n ratio (close to 1), which is expected to be much higher than the proportion of all the genes involved in specific GO term (B/N low, therefore b/n > B/N). In contrast, transcriptional interferences scattered throughout numerous processes (b low) will result in a low b/n ratio (<< 1) and therefore a very weak enrichment (b/n ≈ B/N). Although the various tools developed over time to interrogate gene ontology define their own metrics, the underlying logic is very similar and most of the differences stem from their assessment of statistical robustness.

This has very practical consequences, because the very nature of the transcriptional changes may prevent enrichment analysis to reach statistically significant terms. This explains very well our results, where the lists of DE genes fail to highlight biological contrasts, while our system-level analysis shows that most DE genes are scattered throughout the network. In agreement, alternative tools (R package goProfiles, Sanchez et al., 2016) also failed to show significant differences between datasets (not shown) when considering all DE genes simultaneously. Combining enrichment analysis to system biology proved to be much more resolutive, as restricting the analysis to the subnetworks (*i.e.* sets of functionally connected DE genes co-operating together) increase the b/n ratio, and result in a clear enrichment. Biologically, the terms are related to immune system and neurobiology, and although the influence of TBBPA on neural cells is not new, such impact on the immune system is.

This is not a trivial result, and it may well represent an inherent limitation of toxicogenomics, whereby transcriptional interferences scattered at multiple targets may prevent the biological interpretation of RNA-Seq data. As such, poor contrast in enrichment analyses may not indicate a limited toxicological impact, but rather an analytical consequence of system-wide properties. Luckily, because it is a naive and integrative analysis framework, system biology is perfectly suited to characterize and measure these properties.

### Shared functional content within subnetworks

Although it is based on a similar rationale, our approach is not akin to network propagation, which has been a point of interest in recent years (Barel and Herwig, 2018). In short, network propagation combines experimental data (*e.g*., transcriptomic responses) with molecular interaction knowledge structured in protein-protein interaction or signaling networks. The topology of the network is then used to propagate individual node’s response to nearby nodes throughout the network (including non DE genes), and by that help amplify signal and functionally interpret the experimental data (Barabási and Oltvai, 2004). The underlying postulate is that interacting factors collectively participate to common processes and convey functional perturbations. In this context, interacting non-DE genes could not be used as markers, their role is just limited to enhance the functional information conveyed by DE genes. These approaches are very efficient at identifying sub-modules, improving the functional description of biological processes (enrichment analysis) and identifying novel anti-cancer drug targets (Zecena et al., 2018; Tran and Pham, 2021). For example, Barel and Herwig found different gene sets when comparing the effect of structural analogs of anthracyclines, with very little overlap (Barel and Herwig, 2018). After network propagation (*i.e.,* subnetwork enriched in non-DE neighbors) they found >200 genes in common and a much improved disease pathway enrichment.

In our work, the subnetwork was not built with network propagation. Instead, it was build with more stringent criteria and only incorporate DE genes (non-DE genes being excluded). Nonetheless, given the sheer size of the subnetwork (>100 genes), its shared functional information content is expected to be maximal and there is little doubt that the corresponding biological functions (immune system) are the top molecular phenotypic alterations induced by TBBPA.

We should emphasize that our work did benefit from time course series allowing us to collect a large set of DE genes affected at a time point or another. It is clear that despite a limited overlap of DE genes between experiments, many of them belong to similar pathways and are often neighbors within the network. It is very likely that network propagation carried out on only a single experiment would have reached similar conclusion.

### Altered gene expression induced by TBBPA

Biological networks are highly structured and share a key property: node connectivity follow a power law distribution (“scale free” property (Barabási and Oltvai, 2004)). This imply that most nodes are loosely connected to the network, while a limited number of them are highly connected (“hubs”). Hubs are integration points within networks, and are either convergence points of sensory information or factors controlling a large palette of downstream effectors.

Biological networks are robust against random attacks/perturbations because they statistically affect nodes with low connectivity, and will unlikely impact the function and the structure of the network as a whole. In other words, even with perturbations, nodes remain connected through multiple routes, and affecting one does not prevent signal propagation through alternative paths. In contrast, networks are highly sensitive to targeted attacks/perturbations at hubs, and any compound affecting them is known to have broad and far-reaching impact, resulting in strong toxicity and numerous adverse effects (*e.g.* Piñero et al., 2018; Barel and Herwig 2018). This is well illustrated with cancer cells, which often hijack hub functions and thereby prevent the homeostatic balance of cellular activity (Barabási and Oltvai, 2004), followed by massive functional switches. Therefore, describing the tropism of DE genes for the different network compartments provide important mechanistic informations about the dynamic in place at the scale of the system as a whole.

We found that transcriptome alterations after 4 days treatment are under-represented at hubs and are mainly distributed throughout the network, *i.e*. the most robust component against perturbations. Accordingly, the biological functions affected are fairly-diverse, but with a slight trend on signaling (intracellular calcium, phototransduction) or metabolism (see Supplementary Table S4). This fact, together with the numerous accounts of TBBPA molecular phenotypic alterations (Van der Ven et al., 2008; Dunnick et al., 2015; Parsons et al., 2019), indicate that despite targeting the most robust network compartment, the TBBPA impact is not fully buffered and may be relatively high. This conclusion is not a truism because transcriptome perturbations can be well buffered *in vivo*, even with very potent glucocorticoids (only 38 DE genes in *Xenopus* tailfin after 100 nM corticosterone treatment (Buisine et al., 2021)).

Even if TBBPA has raised concern since 1979 (Inouye et al., 1979), the breadth of its effect remains poorly known. In fact, previous studies reported impact on endocrine systems (thyroid and reproductive) and neurobiology/development causing numerous indirect side effects. Most studies commonly use on a limited set of well-established molecular and cellular markers (Watanabe et al., 2010; Liang et al., 2019b). Very little is known about additional biological processes, and TBBPA toxicity has even been challenged (Schauer et al., 2006; Dong et al., 2021). In this context, our findings of a set of genes respondent to TBBPA, of novel markers of neurological functions and strong transcriptional signature on immune system may help settle this debate.

### A small set of genes respondent to TBBPA as potential new markers

In a toxicological context, the little overlap between time points implies that most DE genes can not be used as markers of TBBPA exposure because expression changes are time-dependent. Fortunately, a small set of genes DE in ≥6 experimental conditions (out of 10 total) providing potential time-independent candidate markers. The best candidate, *Acmsd*, is DE at all time points and TBBPA concentration. A few of these markers are connected to neural biology. We will quickly review their function. *Acmsd* is the only gene DE in all experimental conditions. This gene encodes for an alpha-amino-beta-carboxy-muconate-epsilon-semialdehyde decarboxylase which convert ACMS into AMS (alpha-aminomuconate semialdehyde) and prevent the accumulation of quinolate (QA), a neuronal excitotoxin involved in neurodegenerative disorders. Thus, *Acmsd* is a potential new marker of the effect of TBBPA. Unfortunately, there is no experimental data supporting the molecular mechanism of this connection, as it is required for the development of an adverse outcome pathway (AOP). We note, however that Matsuda et al. (Matsuda et al., 2014) proposed that *Acmsd* gene expression may be linked to the cholesterol metabolism pathway through the regulation of *Srebf2*. In turn, *Srebf2* dysregulation, together with altered cholesterol metabolism, is part of the proposed mode-of-action of TBBPA, leading to the key event “oligodendrocyte hypo-myelination” (Klose et al., 2021). The mechanistic connection between *Acmsd* and *Srebf2* remains an open question.

The other genes are not DE in all conditions but are positive at each time point and thus may represent interesting candidate markers. *Cabp4* encodes a calcium binding protein expressed in bipolar cells in the eyes leading to congenital stationary night blindness if the gene is mutated (Smirnov et al., 2018). *Fthl17a* encodes a ferritin heavy chain like 17 and is expressed in embryonic germ cells (Ruzzenenti et al., 2015). *Dq267100* is a snoRNA whose function remain unknown.

ACP4 is an acid phosphatase deregulated in prostate cancer cell line and in testicular cancer tissues. *Aldoart2* encodes an aldolase fructose-bisphosphate A known to been expressed during development. Deregulation of this gene could be involved in cancer progression. *Lhfpl1* belong to a family of LHFP genes known to be involved in deafness if they are mutated. *Mir6921* is a gene encodes for a miRNA and defect lead to abnormal behaviors, craniofacial anomalies and gene dosage imbalance. For GM20199, very little is known, except that its expression dependent on CREBBP in the context of acute myeloid leukemia (Zimmer et al., 2012).

Two hubs clearly stand out when considering individual subnetworks, VAV1 et BTK, which are DE at all time point but one (BTK, D18). Other hubs significantly differ between time points. The fact that two hubs are found in a subnetwork is not surprising because in scale free networks, connected DE genes pairs are more likely to include hubs and they will tend to accumulate with increasing gene pairs. It is more the fact that they are found consistently even after long treatment duration (4 vs. 28 days), where indirect effects can dominate molecular responses. These two gene products are important factors of B-, T- and NK cells maturation and function (see below). Therefore, the recurrent presence of VAV1 and BTK in subnetworks points to a robust alteration of immune system homeostasis.

### Markers of TBBPA effects on neurological functions

The impact of TBBPA on the nervous system is not new and has been the subject of numerous reports (Nakajima et al., 2009; Chen et al., 2016; Zhu et al., 2018). In a few studies, *in vivo*, its effects have been scored by measuring phenotypic constants rather than by expression changes of a few marker genes: TBBPA impairs nicotinic receptors functions in frontal cortex (Viberg and Eriksson, 2011). It also reduces hypocampus neurogenesis, activate glial activation (without neuronal loss) and reduced BDNF-CREB signaling, resulting in memory loss (Kim et al., 2017). Primary culture of hippocampus neurons treated with TBBPA resulted in the induction of a few markers of apoptosis: increased LDH activity as well as DNA fragmentation and condensation (Szychowski and Wójtowicz, 2016).

*In vitro,* Liang et al., 2019a assessed the impact of TBBPA on neural ectoderm differentiation. They quantified transcriptome alterations by RNA-seq followed by GO terms analysis and extensive RT-qPCR validations*.* Only two neurobiology-relevant GO terms (“nervous system development” (GO:0007399) and “midbrain development” (GO:0030901)) were found to overlap with their very small list of DE genes: *Rfx4*, *Barhl1*, *Cbln1*, *Serf1a*, *Chrna3*. Based on this, they proposed that TBBPA alters axon growth/guidance and neurotransmission.

In this context, our work provides additional data and suggest a wider impact, because several cells types may be affected *in vitro*: microglia, oligodendrocytes and neuronal intermediate progenitor cells. The first DE gene of interest is neuropeptide Y (*Npy*), which participate in numerous physiological processes mainly (but not only) in the nervous system (Rezitis et al., 2022) (Ubuka and Tsutsui, 2022) (Yannielli and Harrington, 2001; Loh et al., 2017). Interestingly, among other things, NPY regulates the secretion of thyrotropin releasing hormone (TRH) by thyrotropin-releasing hormone neurons (Fekete et al., 2001), which may enhance further the action of TBBPA as a disruptor of thyroid hormone signaling (Decherf et al., 2010). The expression of two differentiation markers of multipotent glial progenitor cells (*Ascl1* and *Olig2*) is affected by TBBPA (Szu et al., 2021), together with two transcription factors (*Nkx2-2* and *Sox10*) acting downstream in the cellular differentiation pathway of oligodendrocytes (Sugimori et al., 2008). This is a strong indication that TBBPA impact oligodendrocytes differentiation *in vitro*, well in line with previous report (Klose et al., 2021). Finally, key markers of neurons cells development are also impacted by TBBPA: *Ppp1r17*, *Penk*, *Neurog1* and *Neurog2*. Altered expression of these genes is known translate into various neurological and metabolic diseases (Girskis et al., 2021) (Huang et al., 2014). The expression of other genes involved in various aspects of brain development is also affected: *Tbr1*, *Crym* and *Pax6* (Hevner et al., 2001, 2002) (Kinney and Bloch, 2021) (Georgala et al., 2011; Cvekl and Callaerts, 2017).

### TBBPA impact immune system functions

mESCs can differentiate into virtually any cell type, depending on the experimental setup (culture media, hormone/growth factor supplementation etc). The vast repertoire de developmental trajectories of differentiating mESCs is well illustrated in previous work (Zhou et al., 2010), but also in Liang et al. (2019a). They show expression of gene marker characteristic of each embryonic layer (ectoderm, mesoderm, endoderm), as well as various cell populations: neural cells, cardiomyocytes, thyroid gland, keratinocytes and hepatocytes. The fact that immune cells originate from mesoderm is a first indication that the experimental system they used can generate immune cells. A second indication is that differentiating mESCs into neural cells also produce many additional cells types, including cells expressing collagens, glia, and other immune cells (Briggs et al., 2017). Remarkably, using different differentiation protocols channel cells to alternative developmental trajectories, but the resulting cell populations consistently reach the same differentiation states, including immune cells (Briggs et al., 2017). In addition, mESCs are well known to differentiate into macrophages (Hai et al., 2022), microglia (Beutner et al., 2010), and cells expressing lymphocytes costimulatory molecules (Ling et al., 1998). For these reasons, *in vitro* differentiation of mESCs into neural cells is expected to produce a small proportions of immune cells.

Given the strong emphasis on neural development in Liang et al. (Liang et al., 2019a), the very strong transcriptional signature on immune system was unexpected. Unfortunately, it did not receive as much attention as the nervous system or the hypothalamo-pituitary-gonadal (HPG) axis, and therefore our current understanding of these effects is poor. In this context, providing a list of potential novel endpoints is valuable and further illustrate the strength of combining functional genomics and system biology.

We found that the subnetwork concentrates many DE genes belonging to several immune system pathways. In fact, immune system markers are not limited to the subnetwork but are also found in the non-subnetwork part. Overall, TBBPA affects markers of both lymphoid and myeloid lineages (Alberti-Servera et al., 2017). It is clear, however, that DE genes shared by multiple cell types are expected to have broad effects on immune system.

A limited number of studies reported an impact on the immune system (Watanabe et al., 2010). report enhanced sensitivity to respiratory syncytial viruses after dietary exposure to very high doses of TBBPA (1,700 mg/kg). Exposure with lower doses has no significant effect on spleen weight. *In vitro*, NK cells undergo lysis after exposure to mM scale concentrations of TBBPA (Hurd and Whalen, 2011). At the micro-molar range, TBBPA modulates inflammatory response, and induces cyclooxygenase-2 through pro-inflammatory transcription factors NF-κB and AP-1 (Han et al., 2009).

The main marker used to monitor TBBPA effects on immune system is the production of cytokines. For example, this is the case of tumor necrosis factor alpha (*Tnf-α*), interferon γ (*Ifnγ*) or *Il-4/6/8/24* (Watanabe et al., 2010, 2017; Koike et al., 2013; Park et al., 2014; Almughamsi and Whalen, 2016). In another study Wang et al. (Wang et al., 2019) found that macrophages exposed to TBBPA over-express pro-inflammatory cytokines (*Il-1β*, *Il-6*, and *Tnf-α*), together with a reduced expression of antigen-presenting-related genes (including *Cd86*, DE in our datasets).

Although a few of these markers are DE in our dataset (*e.g*. *Il24*, *Cd86*), we provide a much extended list TBBPA-sensitive genes (85 genes, Figure 4C) relevant for immunity. We acknowledge that some of these genes may be shared by multiple cell types, including non-immune cells, and we will focus below on specific markers of immune cell types defined by Alberti-Servera et al. (2017).

We found two hubs DE in all experimental conditions except one: VAV1 et BTK. Together with EGFR, which gene is also DE, they collectively participate to essential steps of immune cells maturation and functions, both for innate and adaptative responses (Turner and Billadeau, 2002; Strijbis et al., 2013). As expected, the simultaneous genetic inactivation of both *Vav1* and *Btk* leads to severe defect of splenic B cells population, which exhibit a very immature phenotype (Betzler et al., 2022). The splenic micro-architecture and the formation of efficient germinal centers are also affected (Betzler et al., 2022). Such broad effects and strong phenotype illustrate well how biological networks are fragile against targeted attacks at hubs.

We found alterations of general markers of myeloid cells: *Elane*, *Ctsg*, *Fcer1g*, *Ccr2*, *Mpo*, *Csf1r*, *Ctsh*. The two genes *Elane* and *Ctsg* both encode peptidases mediating together the proteolytic cleavage of histone H3 and promote the monocyte to macrophage differentiation (Cheung et al., 2021). *Ccr2* is a chemokine membrane receptor involved in PI3K and JAK-STAT pathways, working together with *Csf1r* to mediate macrophage recruitment and differentiation (Ito et al., 2008; Hume and MacDonald, 2012). Finally, CTSH is a cathepsin BA, a protease involved in innate immune system and MHC antigen presentation.

In term of lymphoid lineage, two markers common to B and T cells are dysregulated: *Il7r*, a membrane receptor playing a critical role for V(D)J recombination, and DNTT, a template-independent DNA polymerase essential for in B and T-cells differentiation and for generating the pool of antibody diversity. There also are four markers specific of B cells population. The function of IGLL1 is connected to signal transduction and the balance between proliferation and differentiation of pre and pro B cells (Lu et al., 2019). The factors CD79A and B are membrane bound immunoglobulin associated proteins critical for signal transduction and the function of B cell antigen receptor (Sakaguchi et al., 1988; Hermanson et al., 1989). BLNK is an intracellular protein that bridges B cells receptor-associated kinase activation with downstream signaling pathways (Pappu et al., 1999). Its function as a mediator of signal transduction is therefore important for B-mediated immune response. It is noteworthy that *Blnk*, *Cd79a* and *Cd79b* form a small “chain” within the subnetwork, meaning that they directly interact with each other and functionally cooperate. The fact that they are dysregulated is predictive of a strong disruption of membrane receptor signaling and intracellular signal transduction by TBBPA in these cells. We found a single maker of T cell population, ZAP70, which is a target of TCR mediated phosphorylation and intracellular signal transduction (Chan et al., 1992).

### A potential for long term TBBPA effects ?

The experimental setup designed by Liang et al. is based on a continuous exposure to 10 or 100 nM TBBPA, with culture media supplemented with TBBPA renewed everyday. Given the numerous direct and indirect effects on developing embryoid body during extended incubation time (up to 28 days), it is difficult to infer any mechanistic insight on the mode of action of TBBPA. The datasets rather reflect the cascading effects of chronic exposure during a differentiation processes *in vitro*. The relevance of TBBPA toxicity for a critical developmental window vs. the whole lifespan depends of the cell population being addressed.

The fact that TBBPA is a neurotoxicant has been questioned because the blood brain barrier (BBB) is a potent molecular filter protecting the central nervous system (Kacew and Hayes, 2020; Zhou et al., 2020; Denuzière and Ghersi-Egea, 2022). Nonetheless, the BBB is slightly leaky at two locations in the central nervous system: the choroid plexus at hypocampus, and the median eminence at the root of the hypothalamus. These serve as sensors of the metabolic state (Goodman and Hajihosseini, 2015) and as discussed above, TBBPA might have a local action there, throughout lifespan. In addition, various pathological contexts are linked to defects in BBB integrity, such as neuroinflammation (Serna-Rodríguez et al., 2022), epilepsy (Oby and Janigro, 2006) and traumatic injuries. In these cases, TBBPA has the potential of broader effects that may not be limited to the developmental window before the establishment of the BBB.

In addition, immune cells are not safeguarded in a restricted territory, and they are scattered in the body and traffic freely through the vascular systems. It is therefore likely that immune cells can be exposed to TBBPA during the whole lifespan. The fact that BTK is a key mediator of innate immunity and inflammation (Weber et al., 2017; Weber, 2021) strongly suggest that TBBPA has the potential to lead to a strong imbalance, which in turn could induce and/or aggravate damages to the BBB integrity (Ní Chasaide and Lynch, 2020), and permeate TBBPA in the central nervous system with more direct effects. Of note, BTK inhibitor share a variety of clinical applications, ranging from treatment of B cell malignancies, to autoimmune diseases and COVID-19 (Zhu et al., 2021; Malekinejad et al., 2022; Rezaei et al., 2022; Weis et al., 2022), which raise the question on whether TBBPA may interfere with these therapeutic actions. This will certainly require dedicated experimental exploration and validation.

## Conclusion

In this work, we used system biology as a complement to functional genomics to characterize the phenotypic effects of TBBPA during the growth and differentiation of mESC. This readily identified a number of integration hotspots within molecular networks (hubs), as well as a collection of gene products functionally interacting together and collectively participating to a limited number of biological processes (subnetworks). In agreement with previous work, we found a clear alteration of markers related to neural functions. Unexpectedly, we also found very strong repression of markers related of the terminal differentiation of several immune cells, suggesting a strong impact of TBBPA on this essential system. This is bad news: not only TBBPA impacts rthe homeostatic balance of several endocrine pathways and neural development, but it also has the potential to affect the main system safeguarding body integrity against aggressors.

## Data Availability

The original contributions presented in the study are included in the article/Supplementary Material, further inquiries can be directed to the corresponding author.

## Supplementary material

Supplementary Data Sheet S1 MA plots of differentially expressed genes in mESCs after TBBPA treatment.

Supplementary Video S1 TBBPA-induced transcriptional changes in differentiating mESCs.

## References

[B1] Abdallah M. A.-E. Pawar G. Harrad S. (2015). Evaluation of 3D-human skin equivalents for assessment of human dermal absorption of some brominated flame retardants. Environ. Int. 84, 64–70. 10.1016/j.envint.2015.07.015 26232142

[B2] Alberti-Servera L. Muenchow L. Tsapogas P. Capoferri G. Eschbach K. Beisel C. (2017). Single-cell RNA sequencing reveals developmental heterogeneity among early lymphoid progenitors. EMBO J. 36, 3619–3633. 10.15252/embj.201797105 29030486 PMC5730887

[B3] Almughamsi H. Whalen M. M. (2016). Hexabromocyclododecane and tetrabromobisphenol A alter secretion of interferon gamma (IFN-γ) from human immune cells. Arch. Toxicol. 90, 1695–1707. 10.1007/s00204-015-1586-6 26302867 PMC4767696

[B4] Anders S. Huber W. (2010). Differential expression analysis for sequence count data. Genome Biol. 11, R106. 10.1186/gb-2010-11-10-r106 20979621 PMC3218662

[B5] Ashburner M. Ball C. A. Blake J. A. Botstein D. Butler H. Cherry J. M. (2000). Gene ontology: Tool for the unification of biology. The gene ontology consortium. Nat. Genet. 25, 25–29. 10.1038/75556 10802651 PMC3037419

[B6] Barabási A.-L. Oltvai Z. N. (2004). Network biology: Understanding the cell’s functional organization. Nat. Rev. Genet. 5, 101–113. 10.1038/nrg1272 14735121

[B7] Barel G. Herwig R. (2018). Network and pathway analysis of toxicogenomics data. Front. Genet. 9, 484. 10.3389/fgene.2018.00484 30405693 PMC6204403

[B8] Betzler A. C. Kieser S. Fiedler K. Laban S. Theodoraki M.-N. Schuler P. J. (2022). Differential requirement of Vav proteins for btk-dependent and –independent signaling during B cell development. Front. Cell. Dev. Biol. 10, 654181. 10.3389/fcell.2022.654181 35281114 PMC8904969

[B9] Beutner C. Roy K. Linnartz B. Napoli I. Neumann H. (2010). Generation of microglial cells from mouse embryonic stem cells. Nat. Protoc. 5, 1481–1494. 10.1038/nprot.2010.90 20725065

[B10] Briggs J. A. Li V. C. Lee S. Woolf C. J. Klein A. Kirschner M. W. (2017). Mouse embryonic stem cells can differentiate via multiple paths to the same state. eLife 6, e26945. 10.7554/eLife.26945 28990928 PMC5648529

[B11] Buisine N. Grimaldi A. Jonchere V. Rigolet M. Blugeon C. Hamroune J. (2021). Transcriptome and methylome analysis reveal complex cross-talks between thyroid hormone and glucocorticoid signaling at Xenopus metamorphosis. Cells 10, 2375. 10.3390/cells10092375 34572025 PMC8468809

[B12] Byrnes K. Blessinger S. Bailey N. T. Scaife R. Liu G. Khambu B. (2022). Therapeutic regulation of autophagy in hepatic metabolism. Acta Pharm. Sin. B 12, 33–49. 10.1016/j.apsb.2021.07.021 35127371 PMC8799888

[B13] Cariou R. Antignac J.-P. Zalko D. Berrebi A. Cravedi J.-P. Maume D. (2008). Exposure assessment of French women and their newborns to tetrabromobisphenol-A: Occurrence measurements in maternal adipose tissue, serum, breast milk and cord serum. Chemosphere 73, 1036–1041. 10.1016/j.chemosphere.2008.07.084 18790516

[B14] Chan A. C. Iwashima M. Turck C. W. Weiss A. (1992). ZAP-70: A 70 kd protein-tyrosine kinase that associates with the TCR ζ chain. Cell. 71, 649–662. 10.1016/0092-8674(92)90598-7 1423621

[B15] Chen J. Tanguay R. L. Xiao Y. Haggard D. E. Ge X. Jia Y. (2016). TBBPA exposure during a sensitive developmental window produces neurobehavioral changes in larval zebrafish. Environ. Pollut. 216, 53–63. 10.1016/j.envpol.2016.05.059 27239688

[B16] Cheung P. Schaffert S. Chang S. E. Dvorak M. Donato M. Macaubas C. (2021). Repression of CTSG, ELANE and PRTN3-mediated histone H3 proteolytic cleavage promotes monocyte-to-macrophage differentiation. Nat. Immunol. 22, 711–722. 10.1038/s41590-021-00928-y 34017121 PMC8159908

[B17] Chin C.-H. Chen S.-H. Wu H.-H. Ho C.-W. Ko M.-T. Lin C.-Y. (2014). cytoHubba: identifying hub objects and sub-networks from complex interactome. BMC Syst. Biol. 8, S11. 10.1186/1752-0509-8-S4-S11 25521941 PMC4290687

[B18] Cho J.-H. Lee S. Jeon H. Kim A. H. Lee W. Lee Y. (2020). Tetrabromobisphenol A-induced apoptosis in neural stem cells through oxidative stress and mitochondrial dysfunction. Neurotox. Res. 38, 74–85. 10.1007/s12640-020-00179-z 32108298

[B121] Cvekl A. Callaerts P. (2017). PAX6: 25th anniversary and more to learn. Exp. Eye Res. 156, 10–21. 10.1016/j.exer.2016.04.017 27126352

[B19] Dankers A. C. A. Roelofs M. J. E. Piersma A. H. Sweep F. C. G. J. Russel F. G. M. van den Berg M. (2013). Endocrine disruptors differentially target ATP-binding cassette transporters in the blood-testis barrier and affect leydig cell testosterone secretion *in vitro* . Toxicol. Sci. 136, 382–391. 10.1093/toxsci/kft198 24014645

[B20] Decherf S. Seugnet I. Fini J.-B. Clerget-Froidevaux M.-S. Demeneix B. A. (2010). Disruption of thyroid hormone-dependent hypothalamic set-points by environmental contaminants. Mol. Cell. Endocrinol. 323, 172–182. 10.1016/j.mce.2010.04.010 20399831

[B21] Denuzière A. Ghersi-Egea J.-F. (2022). Cerebral concentration and toxicity of endocrine disrupting chemicals: The implication of blood-brain interfaces. NeuroToxicology 91, 100–118. 10.1016/j.neuro.2022.04.004 35436567

[B22] Diamandakis D. Zieminska E. Siwiec M. Tokarski K. Salinska E. Lenart J. (2019). Tetrabromobisphenol A-induced depolarization of rat cerebellar granule cells: *Ex vivo* and *in vitro* studies. Chemosphere 223, 64–73. 10.1016/j.chemosphere.2019.02.032 30769291

[B23] Dong M. Li Y. Zhu M. Qin Z. (2021). Tetrabromobisphenol A: A neurotoxicant or not? Environ. Sci. Pollut. Res. Int. 28, 54466–54476. 10.1007/s11356-021-15166-w 34420170

[B24] Dunnick J. K. Morgan D. L. Elmore S. A. Gerrish K. Pandiri A. Ton T. V. (2017). Tetrabromobisphenol A activates the hepatic interferon pathway in rats. Toxicol. Lett. 266, 32–41. 10.1016/j.toxlet.2016.11.019 27914987 PMC5791538

[B25] Dunnick J. K. Sanders J. M. Kissling G. E. Johnson C. L. Boyle M. H. Elmore S. A. (2015). Environmental chemical exposure may contribute to uterine cancer development: Studies with tetrabromobisphenol A. Toxicol. Pathol. 43, 464–473. 10.1177/0192623314557335 25476797 PMC6706771

[B26] Eden E. Navon R. Steinfeld I. Lipson D. Yakhini Z. (2009). GOrilla: A tool for discovery and visualization of enriched GO terms in ranked gene lists. BMC Bioinforma. 10, 48. 10.1186/1471-2105-10-48 PMC2644678 19192299

[B27] Eulaers I. Jaspers V. L. B. Pinxten R. Covaci A. Eens M. (2014). Legacy and current-use brominated flame retardants in the Barn Owl. Sci. Total Environ. 472, 454–462. 10.1016/j.scitotenv.2013.11.054 24300457

[B28] Fekete C. Kelly J. Mihály E. Sarkar S. Rand W. M. Légrádi G. (2001). Neuropeptide Y has a central inhibitory action on the hypothalamic-pituitary-thyroid Axis. Endocrinology 142, 2606–2613. 10.1210/endo.142.6.8207 11356711

[B29] Freitas J. Cano P. Craig-Veit C. Goodson M. L. David Furlow J. Murk A. J. (2011). Detection of thyroid hormone receptor disruptors by a novel stable *in vitro* reporter gene assay. Toxicol. Vitro 25, 257–266. 10.1016/j.tiv.2010.08.013 20732405

[B30] Fu J. Yu M. G. Li Q. Park K. King G. L. (2021). Insulin’s actions on vascular tissues: Physiological effects and pathophysiological contributions to vascular complications of diabetes. Mol. Metab. 52, 101236. 10.1016/j.molmet.2021.101236 33878400 PMC8513152

[B122] Georgala P. A. Carr C. B. Price D. J. (2011). The role of Pax6 in forebrain development. Dev. Neurobiol. 71, 690–709. 10.1002/dneu.20895 21538923

[B31] Girskis K. M. Stergachis A. B. DeGennaro E. M. Doan R. N. Qian X. Johnson M. B. (2021). Rewiring of human neurodevelopmental gene regulatory programs by human accelerated regions. Neuron 109, 3239–3251.e7. e7. 10.1016/j.neuron.2021.08.005 34478631 PMC8542612

[B32] Goodman T. Hajihosseini M. K. (2015). Hypothalamic tanycytes—Masters and servants of metabolic, neuroendocrine, and neurogenic functions. Front. Neurosci. 9, 387. 10.3389/fnins.2015.00387 26578855 PMC4624852

[B33] Guyot R. Chatonnet F. Gillet B. Hughes S. Flamant F. (2014). Toxicogenomic analysis of the ability of brominated flame retardants TBBPA and BDE-209 to disrupt thyroid hormone signaling in neural cells. Toxicology 325, 125–132. 10.1016/j.tox.2014.08.007 25172293

[B34] Hai Q. Han J. Wells S. Smith J. D. (2022). Efficient method to differentiate mouse embryonic stem cells into macrophages *in vitro* . Bio. Protoc. 12, e4318. 10.21769/BioProtoc.4318 PMC8855084 35284603

[B35] Hall S. M. Coulter S. J. Knudsen G. A. Sanders J. M. Birnbaum L. S. (2017). Gene expression changes in immune response pathways following oral administration of tetrabromobisphenol A (TBBPA) in female Wistar Han rats. Toxicol. Lett. 272, 68–74. 10.1016/j.toxlet.2017.03.008 28300664 PMC5425951

[B36] Hamers T. Kamstra J. H. Sonneveld E. Murk A. J. Kester M. H. A. Andersson P. L. (2006). *In vitro* profiling of the endocrine-disrupting potency of brominated flame retardants. Toxicol. Sci. 92, 157–173. 10.1093/toxsci/kfj187 16601080

[B37] Han E. H. Park J. H. Kang K. W. Jeong T. C. Kim H. S. Jeong H. G. (2009). Risk assessment of tetrabromobisphenol A on cyclooxygenase-2 expression via MAP kinase/NF-kappaB/AP-1 signaling pathways in murine macrophages. J. Toxicol. Environ. Health. A 72, 1431–1438. 10.1080/15287390903212873 20077215

[B38] He M.-J. Luo X.-J. Yu L.-H. Liu J. Zhang X.-L. Chen S.-J. (2010). Tetrabromobisphenol-A and hexabromocyclododecane in birds from an E-waste region in South China: Influence of diet on diastereoisomer- and enantiomer-specific distribution and trophodynamics. Environ. Sci. Technol. 44, 5748–5754. 10.1021/es101503r 20666555

[B39] Hermanson G. G. Briskin M. Sigman D. Wall R. (1989). Immunoglobulin enhancer and promoter motifs 5’ of the B29 B-cell-specific gene. Proc. Natl. Acad. Sci. U. S. A. 86, 7341–7345. 10.1073/pnas.86.19.7341 2508087 PMC298057

[B40] Hevner R. F. Miyashita-Lin E. Rubenstein J. L. R. (2002). Cortical and thalamic axon pathfinding defects in Tbr1, Gbx2, and Pax6 mutant mice: Evidence that cortical and thalamic axons interact and guide each other. J. Comp. Neurol. 447, 8–17. 10.1002/cne.10219 11967891

[B41] Hevner R. F. Shi L. Justice N. Hsueh Y. Sheng M. Smiga S. (2001). Tbr1 regulates differentiation of the preplate and layer 6. Neuron 29, 353–366. 10.1016/s0896-6273(01)00211-2 11239428

[B42] Huang C. Chan J. A. Schuurmans C. (2014). “Chapter two - proneural bHLH genes in development and disease,” in *Current Topics in developmental biology* bHLH transcription factors in development and disease. Editor Taneja R. (Massachusetts, United States: Academic Press), 75–127. 10.1016/B978-0-12-405943-6.00002-6 25248474

[B43] Hume D. A. MacDonald K. P. A. (2012). Therapeutic applications of macrophage colony-stimulating factor-1 (CSF-1) and antagonists of CSF-1 receptor (CSF-1R) signaling. Blood 119, 1810–1820. 10.1182/blood-2011-09-379214 22186992

[B44] Hurd T. Whalen M. M. (2011). Tetrabromobisphenol A decreases cell-surface proteins involved in human natural killer (NK) cell–dependent target cell lysis. J. Immunotoxicol. 8, 219–227. 10.3109/1547691X.2011.580437 21623697 PMC3145820

[B45] Inouye B. Katayama Y. Ishida T. Ogata M. Utsumi K. (1979). Effects of aromatic bromine compounds on the function of biological membranes. Toxicol. Appl. Pharmacol. 48, 467–477. 10.1016/0041-008x(79)90430-7 224528

[B46] Ito A. Suganami T. Yamauchi A. Degawa-Yamauchi M. Tanaka M. Kouyama R. (2008). Role of CC chemokine receptor 2 in bone marrow cells in the recruitment of macrophages into obese adipose tissue. J. Biol. Chem. 283, 35715–35723. 10.1074/jbc.M804220200 18977759

[B47] Johnson-Restrepo B. Adams D. H. Kannan K. (2008). Tetrabromobisphenol A (TBBPA) and hexabromocyclododecanes (HBCDs) in tissues of humans, dolphins, and sharks from the United States. Chemosphere 70, 1935–1944. 10.1016/j.chemosphere.2007.10.002 18037156

[B48] Kacew S. Hayes A. W. (2020). Absence of neurotoxicity and lack of neurobehavioral consequences due to exposure to tetrabromobisphenol A (TBBPA) exposure in humans, animals and zebrafish. Arch. Toxicol. 94, 59–66. 10.1007/s00204-019-02627-y 31758204

[B49] Kanehisa M. Goto S. (2000). Kegg: Kyoto Encyclopedia of genes and genomes. Nucleic Acids Res. 28, 27–30. 10.1093/nar/28.1.27 10592173 PMC102409

[B50] Kerdivel G. Blugeon C. Fund C. Rigolet M. Sachs L. M. Buisine N. (2019). Opposite T3 response of ACTG1–FOS subnetwork differentiate tailfin fate in Xenopus tadpole and post-hatching axolotl. Front. Endocrinol. 10, 194. 10.3389/fendo.2019.00194 PMC6454024 31001200

[B51] Kim A. H. Chun H. J. Lee S. Kim H. S. Lee J. (2017). High dose tetrabromobisphenol A impairs hippocampal neurogenesis and memory retention. Food Chem. Toxicol. 106, 223–231. 10.1016/j.fct.2017.05.053 28564613

[B52] Kinney C. J. Bloch R. J. (2021). Μ-crystallin: A thyroid hormone binding protein. Endocr. Regul. 55, 89–102. 10.2478/enr-2021-0011 34020530 PMC9202446

[B53] Kitamura S. Kato T. Iida M. Jinno N. Suzuki T. Ohta S. (2005a). Anti-thyroid hormonal activity of tetrabromobisphenol A, a flame retardant, and related compounds: Affinity to the mammalian thyroid hormone receptor, and effect on tadpole metamorphosis. Life Sci. 76, 1589–1601. 10.1016/j.lfs.2004.08.030 15680168

[B54] Kitamura S. Suzuki T. Sanoh S. Kohta R. Jinno N. Sugihara K. (2005b). Comparative study of the endocrine-disrupting activity of bisphenol A and 19 related compounds. Toxicol. Sci. 84, 249–259. 10.1093/toxsci/kfi074 15635150

[B55] Klose J. Tigges J. Masjosthusmann S. Schmuck K. Bendt F. Hübenthal U. (2021). TBBPA targets converging key events of human oligodendrocyte development resulting in two novel AOPs. ALTEX - Altern. Anim. Exp. 38, 215–234. 10.14573/altex.2007201 33099281

[B56] Koike E. Yanagisawa R. Takigami H. Takano H. (2013). Brominated flame retardants stimulate mouse immune cells *in vitro* . J. Appl. Toxicol. 33, 1451–1459. 10.1002/jat.2809 22972382

[B57] Langmead B. Trapnell C. Pop M. Salzberg S. L. (2009). Ultrafast and memory-efficient alignment of short DNA sequences to the human genome. Genome Biol. 10, R25. 10.1186/gb-2009-10-3-r25 19261174 PMC2690996

[B58] Law R. J. Bersuder P. Allchin C. R. Barry J. (2006). Levels of the flame retardants hexabromocyclododecane and tetrabromobisphenol A in the blubber of harbor porpoises (phocoena phocoena) stranded or bycaught in the U.K., with evidence for an increase in hbcd concentrations in recent years. Environ. Sci. Technol. 40, 2177–2183. 10.1021/es052416o 16646450

[B59] Li Y. Dong M. Xiong Y. Chang Q. Chen X. Fu X. (2022). Effects of postnatal exposure to tetrabromobisphenol A on testis development in mice and early key events. Arch. Toxicol. 96, 1881–1892. 10.1007/s00204-022-03259-5 35230478

[B60] Liang S. Liang S. Yin N. Hu B. Faiola F. (2019a). Toxicogenomic analyses of the effects of BDE-47/209, TBBPA/S and TCBPA on early neural development with a human embryonic stem cell *in vitro* differentiation system. Toxicol. Appl. Pharmacol. 379, 114685. 10.1016/j.taap.2019.114685 31326446

[B61] Liang S. Zhou H. Yin N. Lu Y. Faiola F. (2019b). Embryoid body-based RNA-seq analyses reveal a potential TBBPA multifaceted developmental toxicity. J. Hazard. Mat. 376, 223–232. 10.1016/j.jhazmat.2019.05.030 31129320

[B62] Ling V. Munroe R. C. Murphy E. A. Gray G. S. (1998). Embryonic stem cells and embryoid bodies express lymphocyte costimulatory molecules. Exp. Cell. Res. 241, 55–65. 10.1006/excr.1998.4055 9633513

[B123] Loh K. Zhang L. Brandon A. Wang Q. Begg D. Qi Y. (2017). Insulin controls food intake and energy balance *via* NPY neurons. Mol. Metab. 6, 574–584. 10.1016/j.molmet.2017.03.013 28580287 PMC5444095

[B63] Love M. I. Huber W. Anders S. (2014). Moderated estimation of fold change and dispersion for RNA-seq data with DESeq2. Genome Biol. 15, 550. 10.1186/s13059-014-0550-8 25516281 PMC4302049

[B64] Lu L. Hu J. Li G. An T. (2021). Low concentration Tetrabromobisphenol A (TBBPA) elevating overall metabolism by inducing activation of the Ras signaling pathway. J. Hazard. Mat. 416, 125797. 10.1016/j.jhazmat.2021.125797 33878653

[B65] Lu X. Chu C.-S. Fang T. Rayon-Estrada V. Fang F. Patke A. (2019). MTA2/NuRD regulates B cell development and cooperates with OCA-B in controlling the pre-B to immature B cell transition. Cell. Rep. 28, 472–485. e5. 10.1016/j.celrep.2019.06.029 31291582 PMC6690613

[B66] Malekinejad Z. Baghbanzadeh A. Nakhlband A. Baradaran B. Jafari S. Bagheri Y. (2022). Recent clinical findings on the role of kinase inhibitors in COVID-19 management. Life Sci. 306, 120809. 10.1016/j.lfs.2022.120809 35841979 PMC9278000

[B67] Matsuda H. Sato M. Yakushiji M. Koshiguchi M. Hirai S. Egashira Y. (2014). Regulation of rat hepatic α-amino-β-carboxymuconate-ε-semialdehyde decarboxylase, a key enzyme in the tryptophan- NAD pathway, by dietary cholesterol and sterol regulatory element-binding protein-2. Eur. J. Nutr. 53, 469–477. 10.1007/s00394-013-0547-1 25289390

[B68] Morris S. Allchin C. R. Zegers B. N. Haftka J. J. H. Boon J. P. Belpaire C. (2004). Distribution and fate of HBCD and TBBPA brominated flame retardants in North sea estuaries and aquatic food webs. Environ. Sci. Technol. 38, 5497–5504. 10.1021/es049640i 15575264

[B69] Nakajima A. Saigusa D. Tetsu N. Yamakuni T. Tomioka Y. Hishinuma T. (2009). Neurobehavioral effects of tetrabromobisphenol A, a brominated flame retardant, in mice. Toxicol. Lett. 189, 78–83. 10.1016/j.toxlet.2009.05.003 19463927

[B70] Ní Chasaide C. Lynch M. A. (2020). The role of the immune system in driving neuroinflammation. Brain Neurosci. Adv. 4, 2398212819901082. 10.1177/2398212819901082 32219178 PMC7085916

[B71] Oby E. Janigro D. (2006). The blood–brain barrier and epilepsy. Epilepsia 47, 1761–1774. 10.1111/j.1528-1167.2006.00817.x 17116015

[B72] Pappu R. Cheng A. M. Li B. Gong Q. Chiu C. Griffin N. (1999). Requirement for B Cell linker protein (BLNK) in B cell development. Science 286, 1949–1954. 10.1126/science.286.5446.1949 10583957

[B73] Park H.-R. Kamau P. W. Korte C. Loch-Caruso R. (2014). Tetrabromobisphenol A activates inflammatory pathways in human first trimester extravillous trophoblasts *in vitro* . Reprod. Toxicol. 50, 154–162. 10.1016/j.reprotox.2014.10.005 25461914 PMC4260776

[B74] Parsons A. Lange A. Hutchinson T. H. Miyagawa S. Iguchi T. Kudoh T. (2019). Molecular mechanisms and tissue targets of brominated flame retardants, BDE-47 and TBBPA, in embryo-larval life stages of zebrafish (*Danio rerio*). Aquat. Toxicol. 209, 99–112. 10.1016/j.aquatox.2019.01.022 30763833

[B75] Patil A. Patil A. (2022). CellKb immune: A manually curated database of mammalian hematopoietic marker gene sets for rapid cell type identification. biorxiv 2020, 389890. 10.1101/2020.12.01.389890

[B76] Piñero J. Gonzalez-Perez A. Guney E. Aguirre-Plans J. Sanz F. Oliva B. (2018). Network, transcriptomic and genomic features differentiate genes relevant for drug response. Front. Genet. 9, 412. 10.3389/fgene.2018.00412 30319692 PMC6168038

[B77] Renner H. Becker K. J. Kagermeier T. E. Grabos M. Eliat F. Günther P. (2021). Cell-type-specific high throughput toxicity testing in human midbrain organoids. Front. Mol. Neurosci. 14, 715054. 10.3389/fnmol.2021.715054 34335182 PMC8321240

[B78] Rezaei M. Barati S. Babamahmoodi A. Dastan F. Marjani M. (2022). The possible role of bruton tyrosine kinase inhibitors in the treatment of COVID-19: A review. Curr. Ther. Res. Clin. Exp. 96, 100658. 10.1016/j.curtheres.2021.100658 34931090 PMC8673731

[B79] Rezitis J. Herzog H. Ip C. K. (2022). Neuropeptide Y interaction with dopaminergic and serotonergic pathways: Interlinked neurocircuits modulating hedonic eating behaviours. Prog. Neuropsychopharmacol. Biol. Psychiatry 113, 110449. 10.1016/j.pnpbp.2021.110449 34592387

[B80] Ruzzenenti P. Asperti M. Mitola S. Crescini E. Maccarinelli F. Gryzik M. (2015). The Ferritin-Heavy-Polypeptide-Like-17 (FTHL17) gene encodes a ferritin with low stability and no ferroxidase activity and with a partial nuclear localization. Biochim. Biophys. Acta 1850, 1267–1273. 10.1016/j.bbagen.2015.02.016 25749565

[B81] Saegusa Y. Fujimoto H. Woo G.-H. Inoue K. Takahashi M. Mitsumori K. (2009). Developmental toxicity of brominated flame retardants, tetrabromobisphenol A and 1, 2, 5, 6, 9, 10-hexabromocyclododecane, in rat offspring after maternal exposure from mid-gestation through lactation. Reprod. Toxicol. 28, 456–467. 10.1016/j.reprotox.2009.06.011 19577631

[B82] Sakaguchi N. Kashiwamura S. Kimoto M. Thalmann P. Melchers F. (1988). B lymphocyte lineage-restricted expression of mb-1, a gene with CD3-like structural properties. EMBO J. 7, 3457–3464. 10.1002/j.1460-2075.1988.tb03220.x 2463161 PMC454845

[B83] Sanchez A. Ocana J. Salicru M. (2016). goProfiles: goProfiles: an R package for the statistical analysis of functional profiles.

[B84] Sanders J. M. Coulter S. J. Knudsen G. A. Dunnick J. K. Kissling G. E. Birnbaum L. S. (2016). Disruption of estrogen homeostasis as a mechanism for uterine toxicity in Wistar Han rats treated with tetrabromobisphenol A. Toxicol. Appl. Pharmacol. 298, 31–39. 10.1016/j.taap.2016.03.007 26988606 PMC4825186

[B85] Schauer U. M. D. Völkel W. Dekant W. (2006). Toxicokinetics of tetrabromobisphenol A in humans and rats after oral administration. Toxicol. Sci. 91, 49–58. 10.1093/toxsci/kfj132 16481339

[B86] Serna-Rodríguez M. F. Bernal-Vega S. de la Barquera J. A. O.-S. Camacho-Morales A. Pérez-Maya A. A. (2022). The role of damage associated molecular pattern molecules (DAMPs) and permeability of the blood-brain barrier in depression and neuroinflammation. J. Neuroimmunol. 371, 577951. 10.1016/j.jneuroim.2022.577951 35994946

[B87] Shannon P. Markiel A. Ozier O. Baliga N. S. Wang J. T. Ramage D. (2003). Cytoscape: A software environment for integrated models of biomolecular interaction networks. Genome Res. 13, 2498–2504. 10.1101/gr.1239303 14597658 PMC403769

[B88] Shi Z. Wang Y. Niu P. Wang J. Sun Z. Zhang S. (2013). Concurrent extraction, clean-up, and analysis of polybrominated diphenyl ethers, hexabromocyclododecane isomers, and tetrabromobisphenol A in human milk and serum. J. Sep. Sci. 36, 3402–3410. 10.1002/jssc.201300579 23929782

[B89] Shi Z. Zhang L. Li J. Wu Y. (2018). Legacy and emerging brominated flame retardants in China: A review on food and human milk contamination, human dietary exposure and risk assessment. Chemosphere 198, 522–536. 10.1016/j.chemosphere.2018.01.161 29428767

[B90] Smirnov V. M. Zeitz C. Soumittra N. Audo I. Defoort-Dhellemmes S. (2018). Retinal findings in a patient of French ancestry with CABP4-related retinal disease. Doc. Ophthalmol. 136, 135–143. 10.1007/s10633-018-9629-y 29525873

[B91] Strijbis K. Tafesse F. G. Fairn G. D. Witte M. D. Dougan S. K. Watson N. (2013). Bruton’s Tyrosine Kinase (BTK) and Vav1 contribute to Dectin1-dependent phagocytosis of Candida albicans in macrophages. PLoS Pathog. 9, e1003446. 10.1371/journal.ppat.1003446 23825946 PMC3694848

[B92] Sugimori M. Nagao M. Parras C. M. Nakatani H. Lebel M. Guillemot F. (2008). Ascl1 is required for oligodendrocyte development in the spinal cord. Development 135, 1271–1281. 10.1242/dev.015370 18287202

[B93] Sun H. Shen O.-X. Wang X.-R. Zhou L. Zhen S. Chen X. (2009). Anti-thyroid hormone activity of bisphenol A, tetrabromobisphenol A and tetrachlorobisphenol A in an improved reporter gene assay. Toxicol. Vitro 23, 950–954. 10.1016/j.tiv.2009.05.004 19457453

[B94] Sunday O. E. Bin H. Guanghua M. Yao C. Zhengjia Z. Xian Q. (2022). Review of the environmental occurrence, analytical techniques, degradation and toxicity of TBBPA and its derivatives. Environ. Res. 206, 112594. 10.1016/j.envres.2021.112594 34973196

[B95] Szu J. Wojcinski A. Jiang P. Kesari S. (2021). Impact of the Olig family on neurodevelopmental disorders. Front. Neurosci. 15, 659601. 10.3389/fnins.2021.659601 33859549 PMC8042229

[B96] Szychowski K. A. Wójtowicz A. K. (2016). TBBPA causes neurotoxic and the apoptotic responses in cultured mouse hippocampal neurons *in vitro* . Pharmacol. Rep. 68, 20–26. 10.1016/j.pharep.2015.06.005 26721346

[B97] Tran T.-D. Pham D.-T. (2021). Identification of anticancer drug target genes using an outside competitive dynamics model on cancer signaling networks. Sci. Rep. 11, 14095. 10.1038/s41598-021-93336-z 34238960 PMC8266823

[B98] Trevino A. E. Müller F. Andersen J. Sundaram L. Kathiria A. Shcherbina A. (2021). Chromatin and gene-regulatory dynamics of the developing human cerebral cortex at single-cell resolution. Cell. 184, 5053–5069.e23. e23. 10.1016/j.cell.2021.07.039 34390642

[B99] Turner M. Billadeau D. D. (2002). VAV proteins as signal integrators for multi-subunit immune-recognition receptors. Nat. Rev. Immunol. 2, 476–486. 10.1038/nri840 12094222

[B100] Ubuka T. Tsutsui K. (2022). Neuropeptidergic control of neurosteroids biosynthesis. Front. Neuroendocrinol. 65, 100976. 10.1016/j.yfrne.2021.100976 34999057

[B101] Van der Ven L. T. M. Van de Kuil T. Verhoef A. Verwer C. M. Lilienthal H. Leonards P. E. G. (2008). Endocrine effects of tetrabromobisphenol-A (TBBPA) in Wistar rats as tested in a one-generation reproduction study and a subacute toxicity study. Toxicology 245, 76–89. 10.1016/j.tox.2007.12.009 18255212

[B102] Viberg H. Eriksson P. (2011). Differences in neonatal neurotoxicity of brominated flame retardants, PBDE 99 and TBBPA, in mice. Toxicology 289, 59–65. 10.1016/j.tox.2011.07.010 21820030

[B103] Wang X. Wei L. Zhu J. He B. Kong B. Xue Z. (2019). Environmentally relevant doses of tetrabromobisphenol A (TBBPA) cause immunotoxicity in murine macrophages. Chemosphere 236, 124413. 10.1016/j.chemosphere.2019.124413 31545206

[B104] Watanabe W. Hirose A. Takeshita T. Hashiguchi S. Sakata K. Konno K. (2017). Perinatal exposure to tetrabromobisphenol A (TBBPA), a brominated flame retardant, exacerbated the pneumonia in respiratory syncytial virus (RSV)-infected offspring mice. J. Toxicol. Sci. 42, 789–795. 10.2131/jts.42.789 29142177

[B105] Watanabe W. Shimizu T. Sawamura R. Hino A. Konno K. Kurokawa M. (2010). Functional disorder of primary immunity responding to respiratory syncytial virus infection in offspring mice exposed to a flame retardant, decabrominated diphenyl ether, perinatally. J. Med. Virol. 82, 1075–1082. 10.1002/jmv.21770 20419825

[B106] Weber A. N. R. (2021). Targeting the NLRP3 inflammasome via BTK. Front. Cell. Dev. Biol. 9, 630479. 10.3389/fcell.2021.630479 33718366 PMC7947255

[B107] Weber A. N. R. Bittner Z. Liu X. Dang T.-M. Radsak M. P. Brunner C. (2017). Bruton’s tyrosine kinase: An emerging key player in innate immunity. Front. Immunol. 8, 1454. 10.3389/fimmu.2017.01454 29167667 PMC5682317

[B108] Webster L. Walsham P. Russell M. Neat F. Phillips L. Dalgarno E. (2009). Halogenated persistent organic pollutants in Scottish deep water fish. J. Environ. Monit. 11, 406–417. 10.1039/B815313B 19212601

[B109] Weis T. M. Gutierrez J. Kabel C. C. King A. C. Daley R. J. Stump S. E. (2022). Real-world management of targeted therapies in chronic lymphocytic leukemia. J. Oncol. Pharm. Pract. 28, 1411–1433. 10.1177/10781552221090869 35350909

[B110] Winterhalter C. Widera P. Krasnogor N. (2014). Jepetto: A cytoscape plugin for gene set enrichment and topological analysis based on interaction networks. Bioinformatics 30, 1029–1030. 10.1093/bioinformatics/btt732 24363376 PMC3967109

[B124] Yannielli P. C. Harrington M. E. (2001). Neuropeptide Y in the mammalian circadian system: Effects on light-induced circadian responses. Peptides 22, 547–556. 10.1016/S0196-9781(01)00356-4 11287113

[B111] Yu Y. Yu Z. Chen H. Han Y. Xiang M. Chen X. (2019). Tetrabromobisphenol A: Disposition, kinetics and toxicity in animals and humans. Environ. Pollut. 253, 909–917. 10.1016/j.envpol.2019.07.067 31351299

[B112] Zecena H. Tveit D. Wang Z. Farhat A. Panchal P. Liu J. (2018). Systems biology analysis of mitogen activated protein kinase inhibitor resistance in malignant melanoma. BMC Syst. Biol. 12, 33. 10.1186/s12918-018-0554-1 29615030 PMC5883534

[B113] Zeng Y.-H. Luo X.-J. Tang B. Mai B.-X. (2016). Habitat- and species-dependent accumulation of organohalogen pollutants in home-produced eggs from an electronic waste recycling site in South China: Levels, profiles, and human dietary exposure. Environ. Pollut. 216, 64–70. 10.1016/j.envpol.2016.05.039 27239689

[B114] Zeng Y.-H. Luo X.-J. Zheng X.-B. Tang B. Wu J.-P. Mai B.-X. (2014). Species-specific bioaccumulation of halogenated organic pollutants and their metabolites in fish serum from an E-waste site, South China. Arch. Environ. Contam. Toxicol. 67, 348–357. 10.1007/s00244-014-0040-8 24859045

[B115] Zhou H. Yin N. Faiola F. (2020). Tetrabromobisphenol A (TBBPA): A controversial environmental pollutant. J. Environ. Sci. 97, 54–66. 10.1016/j.jes.2020.04.039 32933740

[B116] Zhou J. Zhang Y. Lin Q. Liu Z. Wang H. Duan C. (2010). Embryoid bodies formation and differentiation from mouse embryonic stem cells in collagen/Matrigel scaffolds. J. Genet. Genomics 37, 451–460. 10.1016/S1673-8527(09)60064-3 20659709

[B117] Zhu B. Zhao G. Yang L. Zhou B. (2018). Tetrabromobisphenol A caused neurodevelopmental toxicity via disrupting thyroid hormones in zebrafish larvae. Chemosphere 197, 353–361. 10.1016/j.chemosphere.2018.01.080 29407805

[B118] Zhu S. Jung J. Victor E. Arceo J. Gokhale S. Xie P. (2021). Clinical trials of the BTK inhibitors ibrutinib and acalabrutinib in human diseases beyond B cell malignancies. Front. Oncol. 11, 737943. 10.3389/fonc.2021.737943 34778053 PMC8585514

[B119] Zhu X. Gerstein M. Snyder M. (2007). Getting connected: Analysis and principles of biological networks. Genes. Dev. 21, 1010–1024. 10.1101/gad.1528707 17473168

[B120] Zimmer S. N. Lemieux M. E. Karia B. P. Day C. Zhou T. Zhou Q. (2012). Mice heterozygous for CREB binding protein are hypersensitive to γ-radiation and invariably develop myelodysplastic/myeloproliferative neoplasm. Exp. Hematol. 40, 295–306. e5. 10.1016/j.exphem.2011.12.004 22198154 PMC3402047

