# Supplementary figures and images for: Tetrabromobisphenol A effects on differentiating mouse embryonic stem cells reveals unexpected impact on immune system
Source: Front Genet. 2022 Oct 25;13:996826. doi: 10.3389/fgene.2022.996826 (PMC9640982; doi:10.3389/fgene.2022.996826)

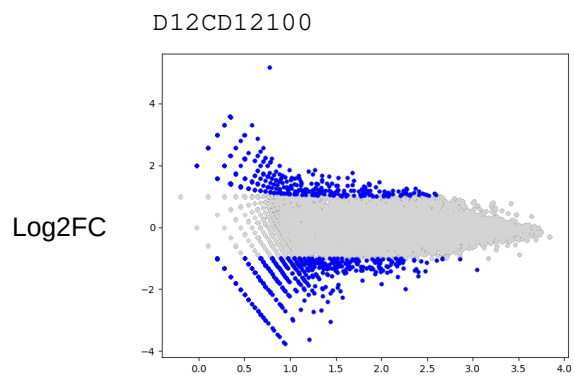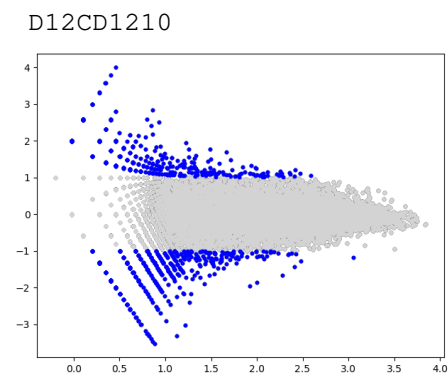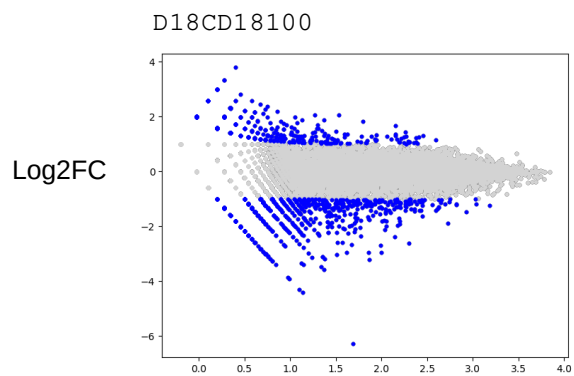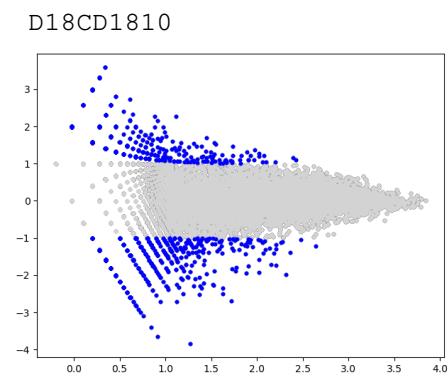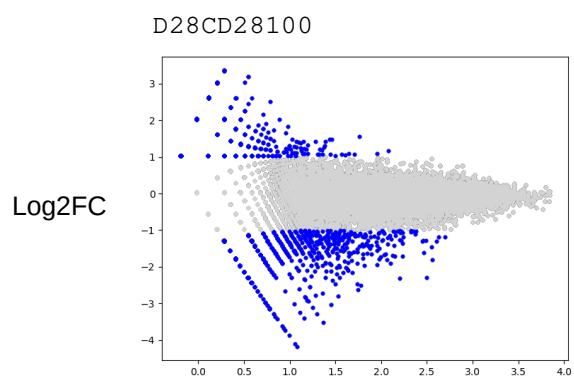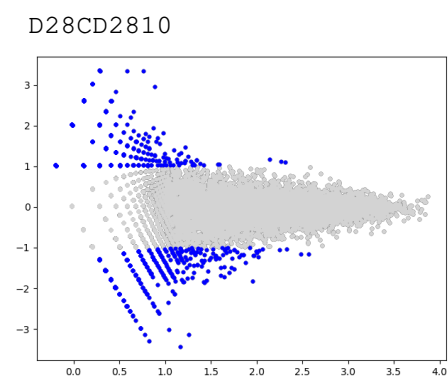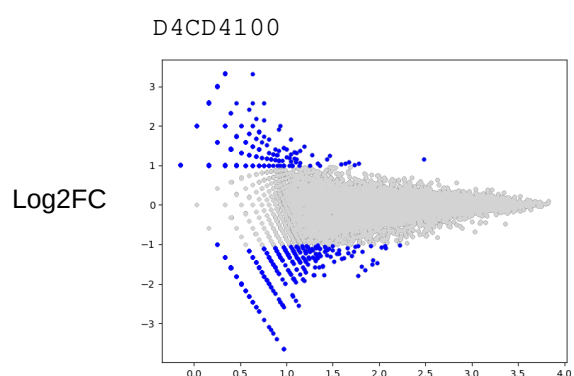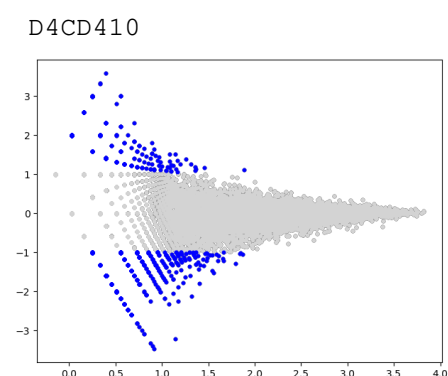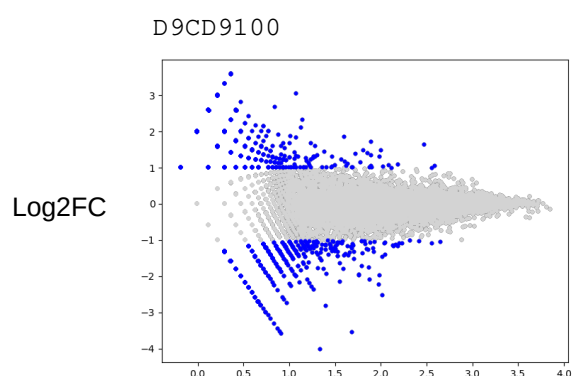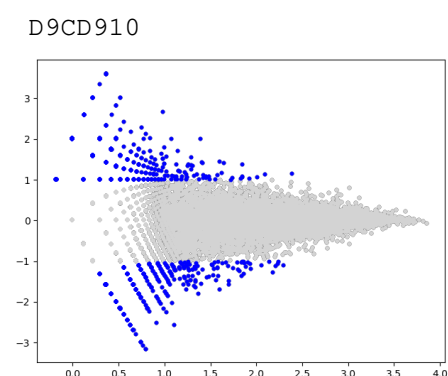

Read count (log10)

Read count (log10)

Supplement: Supplementary file 1 [file DataSheet1.PDF]
